# Gonococcal Mimitope Vaccine Candidate Forms a Beta-Hairpin Turn and Binds Hydrophobically to a Therapeutic Monoclonal Antibody

**DOI:** 10.1021/jacsau.4c00359

**Published:** 2024-07-05

**Authors:** Peter T. Beernink, Cristina Di Carluccio, Roberta Marchetti, Linda Cerofolini, Sara Carillo, Alessandro Cangiano, Nathan Cowieson, Jonathan Bones, Antonio Molinaro, Luigi Paduano, Marco Fragai, Benjamin P. Beernink, Sunita Gulati, Jutamas Shaughnessy, Peter A. Rice, Sanjay Ram, Alba Silipo

**Affiliations:** † Department of Pediatrics, University of California San Francisco, 5700 Martin Luther King Jr. Way, Oakland, California 94609, United States; ‡ Department of Chemical Sciences, University of Naples Federico II, Via Cintia 4, 80126 Naples, Italy; § Department of Chemistry, University of Florence, Via della Lastruccia 13, 50019 Sesto Fiorentino, Italy; ∥ National Institute for Bioprocessing Research and Training, Foster Avenue, Mount Merrion, Blackrock, Co., Dublin A94 X099, Ireland; ⊥ Diamond Light Source, Didcot, OX11 0DE Oxfordshire, England, United Kingdom; # School of Chemical and Bioprocess Engineering, University College Dublin, Belfield Dublin 4, Ireland; ∇ Department of Infectious Diseases and Immunology, University of Massachusetts Chan Medical School, 364 Plantation St, Worcester, Massachusetts 01605, United States

**Keywords:** monoclonal antibody, *Neisseria gonorrhoeae*, gonorrhea, mimitope, peptide, therapeutic, vaccine

## Abstract

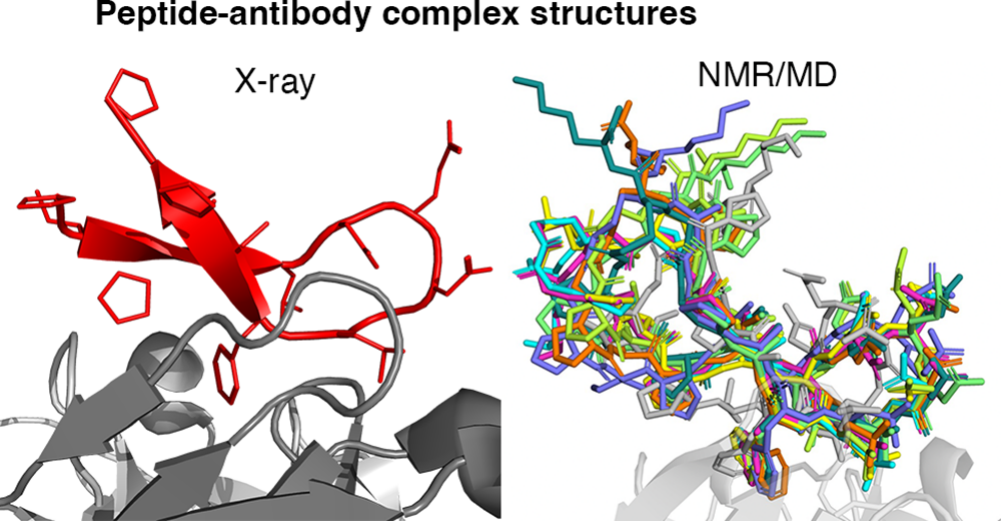

The spread of multidrug-resistant strains of *Neisseria gonorrhoeae*, the etiologic agent of gonorrhea, represents a global health emergency. Therefore, the development of a safe and effective vaccine against gonorrhea is urgently needed. In previous studies, murine monoclonal antibody (mAb) 2C7 was raised against gonococcal lipooligosaccharide (LOS). mAb 2C7 elicits complement-dependent bactericidal activity against gonococci, and its glycan epitope is expressed by almost every clinical isolate. Furthermore, we identified a peptide, cyclic peptide 2 (CP2) that mimicked the 2C7 LOS epitope, elicited bactericidal antibodies in mice, and actively protected in a mouse vaginal colonization model. In this study, we performed structural analyses of mAb 2C7 and its complex with the CP2 peptide by X-ray crystallography, NMR spectroscopy, and molecular dynamics (MD) simulations. The crystal structure of Fab 2C7 bound to CP2 showed that the peptide adopted a beta-hairpin conformation and bound the Fab primarily through hydrophobic interactions. We employed NMR spectroscopy and MD simulations to map the 2C7 epitope and identify the bioactive conformation of CP2. We also used small-angle X-ray scattering (SAXS) and native mass spectrometry to obtain further information about the shape and assembly state of the complex. Collectively, our new structural information suggests strategies for humanizing mAb 2C7 as a therapeutic against gonococcal infection and for optimizing peptide CP2 as a vaccine antigen.

## Introduction

*Neisseria gonorrhoeae* is a leading cause of sexually transmitted infections, which include urethritis, cervicitis, pharyngitis, locally invasive (epididymitis and salpingo-oophritis) and disseminated infections, and in rare cases, meningitis and endocarditis.^1^ Infections in women are frequently asymptomatic, which augments the spread of bacteria among populations. Further, strains have emerged that are resistant to multiple antibiotics, which have the potential to lead to untreatable infections. Thus, the development of vaccines and novel therapeutics, including nonantibiotic approaches, is a high priority. A number of gonococcal surface proteins have been reported to elicit serum bactericidal activity (reviewed in reference (2).) These include Surface-exposed lysozyme inhibitor of C-type lysozyme (SliC) conjugated to virus-like particles (VLPs),^3^ methionine-binding protein (MetQ),^4^ Neisserial heparin-binding antigen (NHBA),^5^ multidrug efflux transporter (E subunit) MtrE,^6^ macrophage infectivity potentiator (MIP),^7^ and adhesin complex protein (ACP).^8^ Immunization with MetQ or MtrE adjuvanted with CpG also was shown to reduce bacterial burden in the mouse vaginal colonization model of gonorrhea.^9,10^ Both gonococcal and meningococcal outer membrane vesicles are also being evaluated for efficacy against gonorrhea (reference (11) and clinical trials NCT04350138, NCT04415424 (meningococcal OMV), and NCT05630859 (gonococcal OMV)).

Gonococcal lipooligosaccharide (LOS) is the most abundant component of the outer membrane. Antibodies against LOS can elicit complement-dependent bacterial killing, and therefore, LOS has been pursued as a vaccine candidate (reviewed in ref (12)). A murine anti-LOS monoclonal antibody (mAb) 2C7 was isolated almost four decades ago.^13,14^ 2C7 recognizes an LOS epitope that comprises lactose from heptose (Hep) II; lactose from HepI or GlcNAc may also contribute to the 2C7 epitope (see Supporting Information, Scheme S1). This mAb reacted with 94% of 68 gonococcal strains examined in the cervical secretions of women^15^ and 100% of 75 minimally passaged clinical isolates of gonococci from a sexually transmitted infections clinic in Nanjing, China.^16^ Murine mAb 2C7 and its human IgG1 chimeric variant both decreased the duration and burden of gonococcal colonization in the mouse vaginal model of gonorrhea.^17,18^ Further, gonococci that lack the 2C7 LOS epitope suffer a significant colonization defect in mice, suggesting that the 2C7 LOS epitope is a key virulence factor.^16,17^ Thus, resistance to antibodies through the loss of expression of the 2C7 LOS epitope, if it were to occur, would confer a substantial fitness cost to gonococci. Taken together, these data suggest that antibodies against the 2C7 LOS epitope could constitute an effective vaccine or therapeutic strategy against gonococcal infections.

An anti-idiotype antibody against mAb 2C7 elicited antibodies against the 2C7 LOS epitope in mice and rabbits,^15^ which suggested that a peptide might also mimic the LOS epitope.^15^ Therefore, a random peptide display library was used to identify a peptide, designated as PEP1, which bound mAb 2C7. When formulated as an octameric multiple antigenic peptide (MAP), it decreased bacterial burden in the mouse vaginal model of gonococcal infection.^17,19^ Subsequently, PEP1 was covalently cyclized to yield CP2 and configured as a tetramer (designated tetra-MAP CP2 or TMCP2). TMCP2 adjuvanted with glucopyranosyl lipid A-stable emulsion (GLA-SE) elicited bactericidal antibodies and hastened clearance of gonococci from the vaginas of mice.^20^

The objective of this study was to perform biophysical studies to probe the molecular interactions between the peptide mimic of the gonococcal LOS epitope (monomeric CP2 or the tetrameric vaccine candidate TMCP2) and its cognate mAb 2C7 or its recombinant Fab fragment. For most of the solution biophysical studies, we used CP2 or TMCP2 and intact IgG mAb 2C7; for crystallographic studies, we used monomeric CP2 and Fab 2C7 to avoid the conformational variability present in TMCP2 or mAb 2C7. Through X-ray crystallography, NMR, and molecular dynamics (MD) simulations, we determined that a central region of the peptide-mediated hydrophobic and polar interactions with the 2C7 antibody, primarily with the heavy chain. Details of the binding energetics were revealed by isothermal titration calorimetry (ITC) experiments. Native mass spectrometry (MS) and small-angle X-ray scattering (SAXS) provided further information about the assembly states and shape of the antibody-peptide complex. Collectively, our studies provide a structural basis for further humanization of 2C7 as a therapeutic antibody and for optimization of the cyclic peptide or its multimers as a gonococcal vaccine candidate.

## Results

### Analysis of TMCP2 and Its Complexes with mAb/Fab 2C7

**Figure 1:**
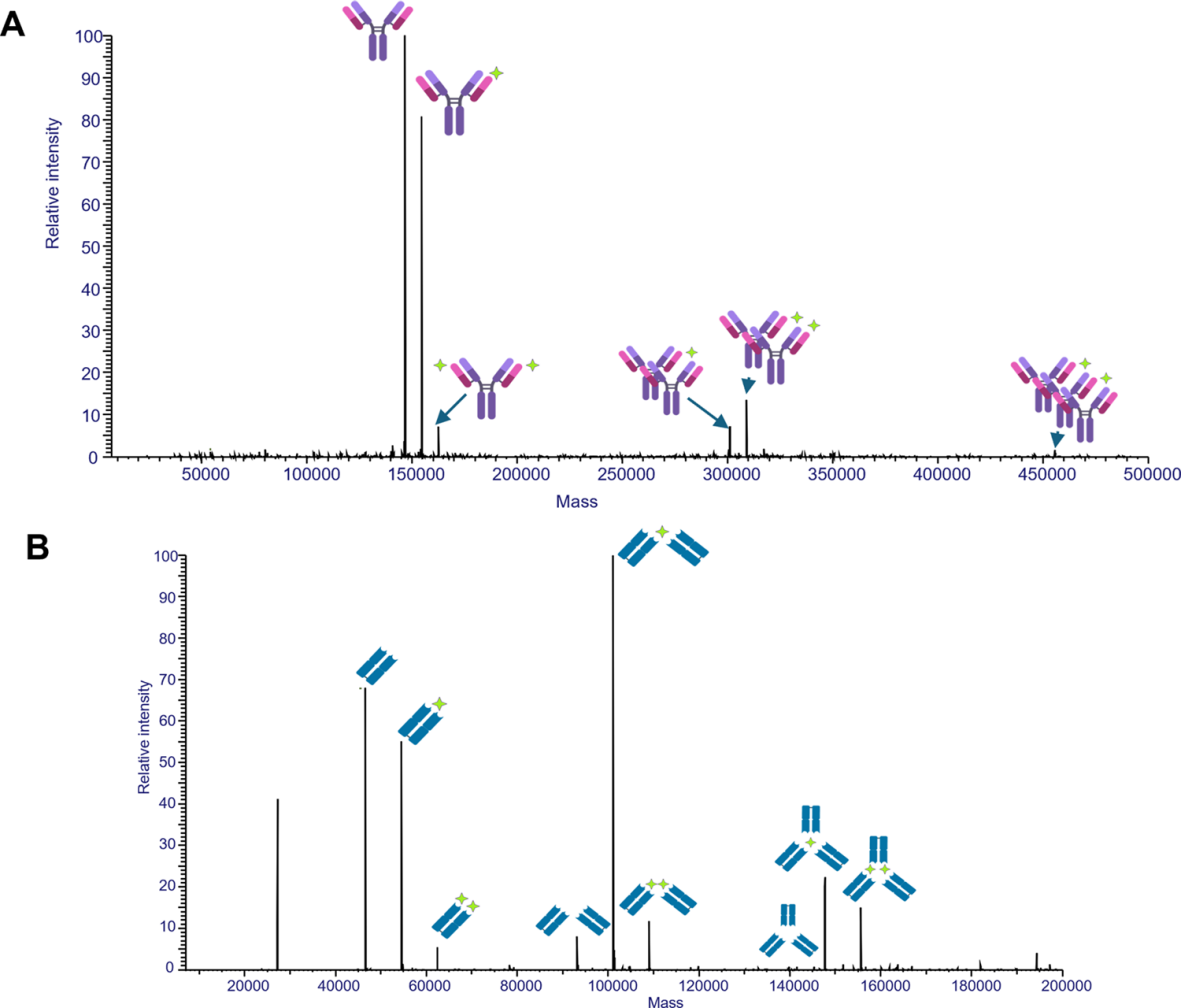
Antibody 2C7 and TMCP2 complexes were detected by native mass spectrometry (MS). (A) Deconvoluted spectrum of the SEC-MS analysis performed on a sample containing mAb 2C7 (violet) and TMCP2 (green) in a 1:4 molar ratio. (B) Deconvoluted spectrum of the SEC-MS analysis performed on Fab 2C7 (blue) and TMCP2 in a 1:4 molar ratio.

We analyzed tetrameric TMCP2 by reversed-phase chromatography-MS to determine its purity and to obtain its average mass. This analysis identified a single peak (99.5% purity, Figure S1A) that, after deconvolution of the associated mass spectrum, gave a monoisotopic mass of 7897.790 Da (average mass 7902.81 Da) (Figure S1B), consistent with previous results.^20^ Next, we analyzed mAb 2C7 and its Fab fragment in the absence and presence of TMCP2 (molar ratio 1:4). SEC-UV chromatograms (Figure S2) showed a clear shift of peaks to shorter elution times, demonstrating the formation of larger antibody–antigen complexes. Finally, we investigated the interactions between mAb 2C7 or its Fab fragment and the TMCP2 tetramer by native MS. This technique preserves protein and ligand interactions^21^ and, thus, can be used to measure the mass shift caused by the interaction between antibody and ligand. Upon deconvolution of the associated mass spectra, we observed one molecule of mAb 2C7 alone or complexed with 1 or 2 molecules of TMCP2 (Figure 1A, first three peaks). The experimental masses were 146,545.34, 154,447.80, and 162,348.46 *m*/*z*, respectively, where each additional TMCP2 bound resulted in a mass shift of ∼7902 Da). In addition, the TCMP2 tetramer promoted multimerization of the mAb; for example, two mAbs formed a complex with one or two molecules of TMCP2 (Figure 1A, fourth and fifth peaks); and three mAbs formed a complex with two molecules of TMCP2 (Figure 1A, sixth peak). Similarly, one Fab interacted with one molecule of TMCP2; two Fabs interacted with one or two molecules of TMCP2, and three Fabs interacted with one or two molecules, (Figure 1B). The presence of nonspecific aggregates in native MS experiments, such as two Fabs interacting with two molecules of TCMP2 (Figure 1B), was possibly due to small amounts of aggregates present in SEC-UV traces (Figure S2).

### NMR-Derived Binding Epitope

**Figure 2:**
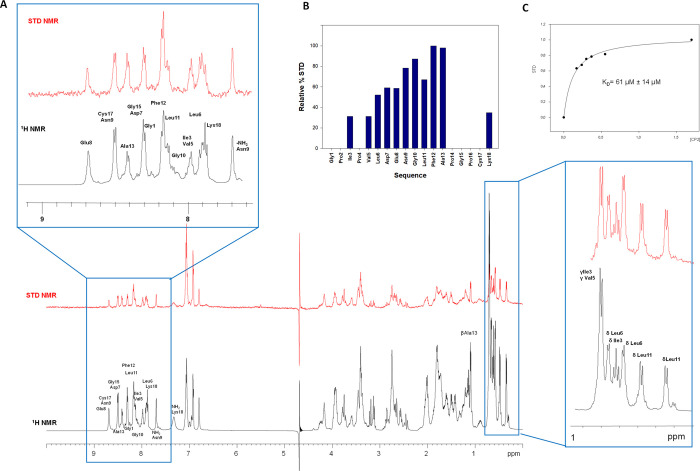
STD NMR spectroscopy. (A) STD NMR (red) and off-resonance (black) spectra of the mixture of mAb and CP2. Specific binding is observed due to the differences in the relative intensities and multiplicities of several signals, as highlighted in the aromatic and aliphatic regions. (B) Relative STD intensities for NH protons. The relative degree of saturation for the individual protons is normalized to the largest STD signal, the Phe12 HN, set to 100%, to evaluate the STD effects. (C) Titration of CP2 into a solution of mAb to calculate *K*_D_ via STD NMR.

We mapped the CP2 epitope by saturation transfer difference (STD) NMR spectroscopy, which allowed us to identify the amino acid residues of the peptide that comprised the binding interface with mAb 2C7^22−25^ (Figure 2). The spectral region corresponding to the amide protons was well resolved and was used to detect the peptide residues in the closest proximity to mAb 2C7 and the relative contributions of their interactions. The strongest signals corresponded to CP2 residue Phe12, suggesting it was the main contact with the antibody; therefore, we used it to normalize the STD enhancements for the other peptide residues (Figure 2B). STD binding data indicated that other CP2 residues, such as Asn9, Gly10, Leu11, and Ala13, whose amide STD relative intensities varied from 68 to 97%, also contributed to the interaction with the mAb. Farther upfield, spectral overlap impeded direct analysis of the intensity of most of the proton resonances. However, in the aliphatic region of the STD spectrum, the terminal γ-methyl groups of Ile3 and Val5, and the δ-methyl groups of Leu6, Ile3, and Leu11 showed well-resolved signals upon binding the mAb. At a lower field, around 7 ppm, the NMR saturation transfer also showed high intensities for the aromatic protons of residue Phe12, which were well amplified in the spectrum, further indicating its proximity to the mAb (Figure 2A). Collectively, these NMR binding data defined the interacting epitope of CP2, with the central region comprising residues 9 to 13 playing a key role in mAb binding. In addition, STD NMR titration experiments yielded an apparent equilibrium dissociation constant (*K*_D_) of 61 μM based on a fit to a one-site Langmuir binding model. The binding isotherm based on the STD amplification factor (STD-AF) values of the methyl signals upon binding to 2C7 is shown in Figure 2C. We further characterized the antibody–peptide interaction using WaterLOSGY,^26^ Carr–Purcell–Meiboom–Gill (CPMG) relaxation-time-edited,^27,28^ and 1D NH chemical shift perturbation (Figure S3).

**Figure 3:**
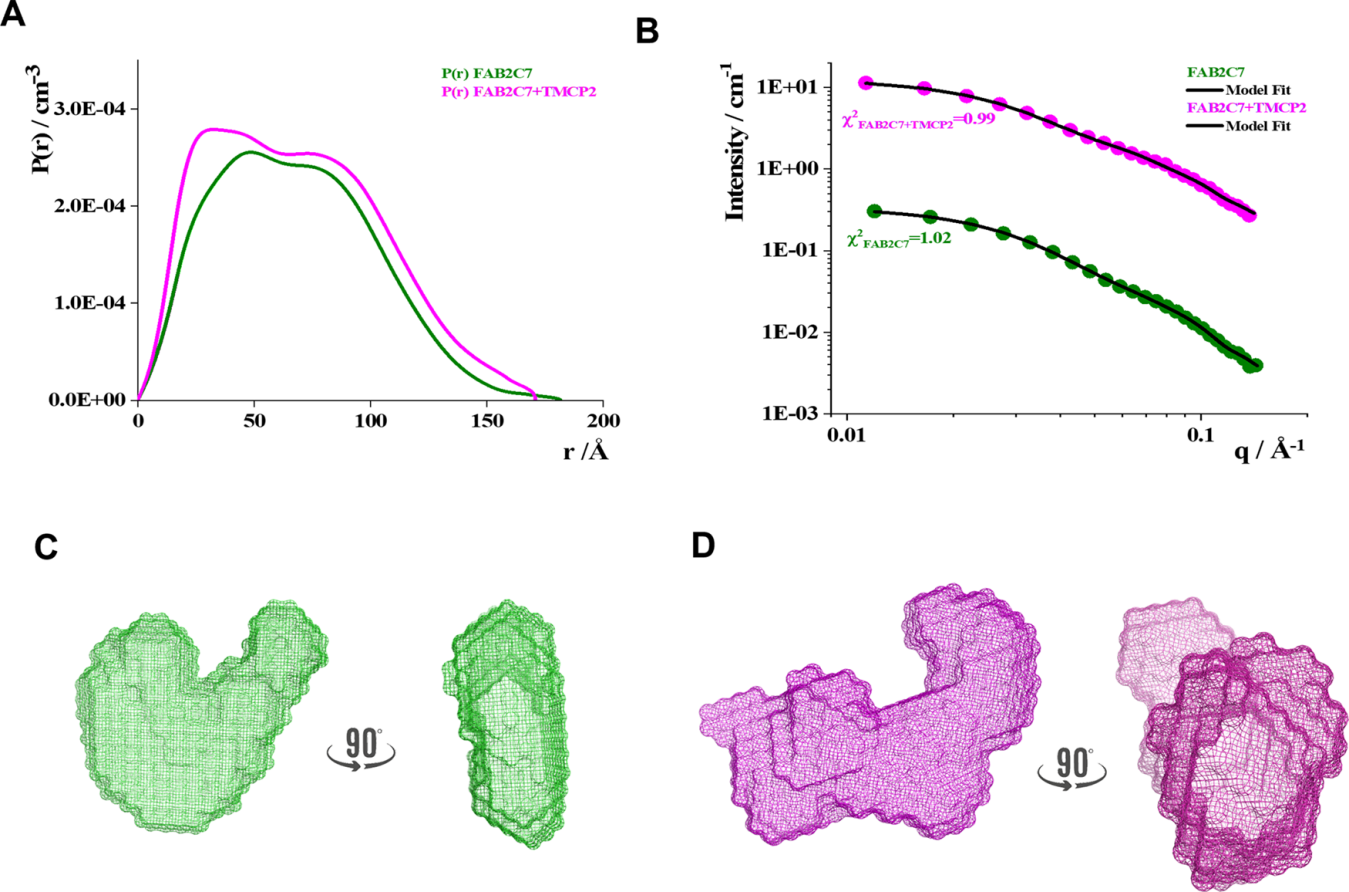
SAXS experiments. (A) Pair correlation function, *P*(*r*). (B) Fitted SAXS profiles were obtained from DAMMIN ab initio reconstruction. (C) Three-dimensional (3D) model obtained for Fab 2C7 alone. (D) 3D model obtained for Fab 2C7 bound to TMCP2 tetramer.

We investigated the molecular interaction between the tetrameric peptide TMCP2 and mAb 2C7 using other ligand-based NMR approaches, including pulsed-field-gradient-stimulated echo (PFG-STE) experiments (Figure S4). These experiments allowed us to calculate the diffusion coefficients of the peptide in the free and bound states from which hydrodynamic radii of 1.4 nm for the tetrapeptide and 3.8 nm for the mAb 2C7-TMCP2 complex were derived. We also conducted SAXS experiments to estimate the shape, conformation, and assembly state of Fab 2C7 upon binding to the TMCP2 tetramer in solution (Figures 3 and S5). SAXS profiles and the derived pair correlation (*P*(*r*)) functions provided evidence for a change in the shape of Fab upon binding TMCP2 (Table 1). An ab initio shape reconstruction of the complex indicated a transition from the free state of Fab 2C7 to its bound state with TMCP2 (Figure 3C, D). Consistency of the SAXS data was shown by comparing the *R*_g_ values obtained from the Guinier analysis (Figure S5) with those from a *P*(*r*) analysis^29^ (Table 1). Moreover, the *q*_min_*R*_g_ obtained from Guinier analysis was <1.3, indicating the goodness of the selected *q* range.

**Table 1:** Guinier Analysis and *P*(*r*) Parameters from SAXS Experiments^a^

sample	Guinier analysis	*P*(*r*) analysis
Fab 2C7	*q*_min_ = 0.0119 Å^–1^	*R*_g_ = 56.2 ± 0.3 Å
	*R*_g_ = 55.9 ± 0.1 Å	
Fab 2C7 + TMCP2	*q*_min_ = 0.0109 Å^–1^	*R*_g_ = 54.8 ± 0.4 Å
	*R*_g_ = 54.6 ± 0.1 Å	

a *q*_min_, minimum scattering vector; *P*(*r*), pair correlation function; *R*_g_, radius of gyration.

### ITC Analysis

We used isothermal titration calorimetry (ITC) to investigate the interaction between mAb 2C7 and CP2 (Figure S6). The data were fitted by using a one-site binding model to derive the thermodynamic parameters and the affinity constant. The binding stoichiometry was 1.89, indicating that one molecule of mAb bound two CP2 peptides,^30^ which is expected for an IgG antibody with two antigen-binding sites. The interaction between mAb 2C7 and CP2 was an exothermic process (Δ*G* < 0); the reaction was both enthalpically and entropically driven, with a negative enthalpy (Δ*H* = −7.17 kJ/mol) and a favorable entropic contribution (−*T*Δ*S* = −18.9 kJ/mol) (Figure S6). These results can be attributed to the hydrogen bonds and hydrophobic interactions at the mAb-CP2-binding interface. The derived *K*_D_ value was 23 μM, which was slightly lower than that obtained by STD NMR experiments (61 μM). Experimental *K*_D_ values calculated by STD NMR are generally greater than or equal to the true thermodynamic values since factors such as STD saturation time, ligand residence time in the complex, and signal intensity affect the accumulation of saturation in the free ligand by processes closely related to fast protein–ligand rebinding and longitudinal relaxation of the ligand signals.^31^

### Crystal Structure of Chimeric Fab 2C7 and Its Complex with CP2

**Figure 4:**
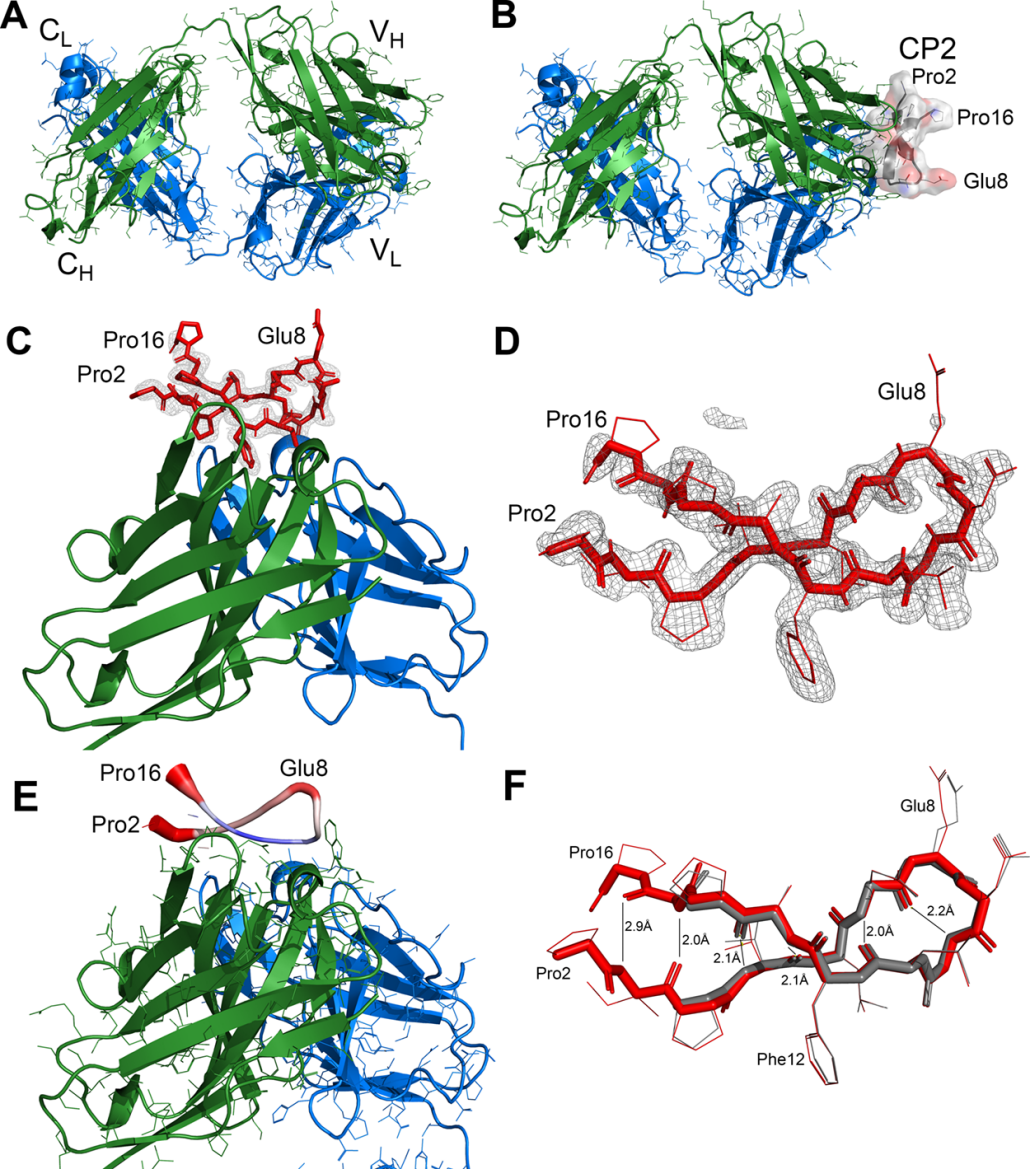
Crystal structures of Fab and the Fab-peptide complex. (A) Fab 2C7 alone (chains A and B). The heavy chain is shown in green, and the light chain is shown in blue. Constant region domains are labeled *C*_H_ and *C*_L_, respectively, and variable region domains are labeled *V*_H_ and *V*_L_. (B) Similar view of Fab 2C7 (chains A and B) in a complex with cyclic peptide 2 (CP2; chain F). CP2 is shown as a surface rendering, colored by atom type (C, gray; N, blue, O, red). (C) View of the variable regions and bound peptide, with a shake-omit electron density map (mFo-DFc) calculated without peptide atoms (contour level 2.5 sigma). CP2 residues 1, 17, and 18 were not visible in the electron density map. (D) Shake-omit map showing a closer view of peptide CP2 from the Fab 2C7 complex. (E) View similar to panel C with CP2 shown in putty representation. The diameter and color of the tube represent the thermal- (B-) factors. Larger diameter and red coloring indicate higher B-factors and smaller diameter and blue color depicts lower B-factors. (F) Hydrogen (H) bonds in the beta-hairpin structure of CP2. The two copies of CP2 (chain F, red; chain E, gray) are superimposed, and the backbone hydrogen bonding distances between nonhydrogen atoms are shown. Chain F had clear electron density for residues 2–16 and chain E had density for residues 4–14. Measurements are based on chain F.

Additional insights into the structural features of the recognition of CP2 by Fab 2C7 were obtained by X-ray crystallography. First, we determined the crystal structure of Fab 2C7 alone by molecular replacement (MR). The space group was P1 and there were two copies of the Fab in the asymmetric unit (heavy chains A/C and light chains B/D). We refined the structure at 1.70 Å resolution to a working R factor (R-work) of 0.177 and a free R factor (R-free) of 0.216 with good geometry. The data collection and refinement statistics are listed in Table S1. The electron density maps were clear and continuous for most of the Fab, including the complementarity-determining region (CDR) loops. There was no electron density (2mFo-DFc) for several loops in the constant region of the heavy chain, including residues 155–160 in both copies A and C, and 216–220 in chain C. A cartoon representation of the Fab structure is shown in Figure 4A.

We determined the structure of the Fab-peptide complex (Figure 4B) by MR with the atomic coordinates of the Fab alone. Before building the peptide model, a difference electron density map (mFo-DFc) showed clear and continuous density (3.0 sigma) in proximity to the CDR loops, which we interpreted to be the peptide. We refined a model of the complex containing most of the peptide residues at 1.65-Å resolution to an R-work of 0.189 and an R-free of 0.234 (Table S1). The refined structure revealed that the peptide adopts a beta-hairpin conformation that is bound in a groove formed by the heavy and light chains of the Fab. One noteworthy result was that the cyclized ends of the peptide were disordered in the structure. Based on a superposition of the Fab with the Fab-peptide complex, the conformation of the Fab did not change substantially upon binding of CP2 (Figure S7A).

An electron density map (2mFo-DFc) calculated at the end of refinement exhibited a clear electron density for nearly all of the Fab and peptide residues. The exceptions included two loops in the heavy chain of the Fab-peptide complex, which had no electron density and were presumably disordered. One loop comprising residues 155 to 161 in the heavy chains (A, C) was also disordered in the structure of Fab 2C7 alone. Another loop containing residues 216 to 220 was disordered in one of the heavy chains (C) in the structure of the complex but ordered in that of the Fab alone. There was interpretable density for peptide residues 4–14 in chain E and 2–16 in chain F. Interestingly, there was no electron density for terminal residues 1 and 17 of the peptide, the thioether bond between them, or for the side chains of residues Glu8 and Pro16. The lack of density for these regions of the peptide was apparent in a shake-omit map (mFo-DFc), which was calculated excluding the peptide (Figure 4C, D). Analysis of the thermal (B) factors of the peptide indicated that the lowest mobility was between residues 9 and 13 (Figure 4E), which coincided with the residues with the largest amide proton STD effects (Figure 2B), and also buried surface areas and solvation energies calculated with PISA (see below).

Finally, we analyzed the interactions of the complex using the Protein Interfaces, Surfaces, and Assemblies (PISA) server.^32^ The buried interaction surface was ∼570 Å^2^, most of which was mediated by the heavy chain (∼480 Å^2^; Table S2). Similarly, the solvation energy calculated for the heavy chain was more negative than that for the light chains (Table S2). PISA identified four hydrogen bonds between the Fab heavy chain and the peptide, which were present in both copies in the asymmetric unit (Table S3), and none between the light chain and the peptide. Within the peptide, there were four and six hydrogen bonds between backbone amide and carbonyl oxygen atoms in the two copies (E and F, respectively), which is characteristic of a beta-hairpin conformation (Figure 4F). The two additional H-bonds in chain F were observed because of electron density for residues 2, 3, 15, and 16 that was not present in chain E. The conformation of the ordered region of the peptide was nearly identical in the two copies (Figure 4F). There were ordered water molecules that bound both the Fab and the peptide: seven in the peptide F chain and six in the E chain (Table S4 and Figure S7B). Of these, two water molecules formed a bridge between corresponding atoms in both copies of the Fab in the asymmetric unit.

### Fab 2C7-CP2 Interactions and Bound Conformation of CP2

**Figure 5:**
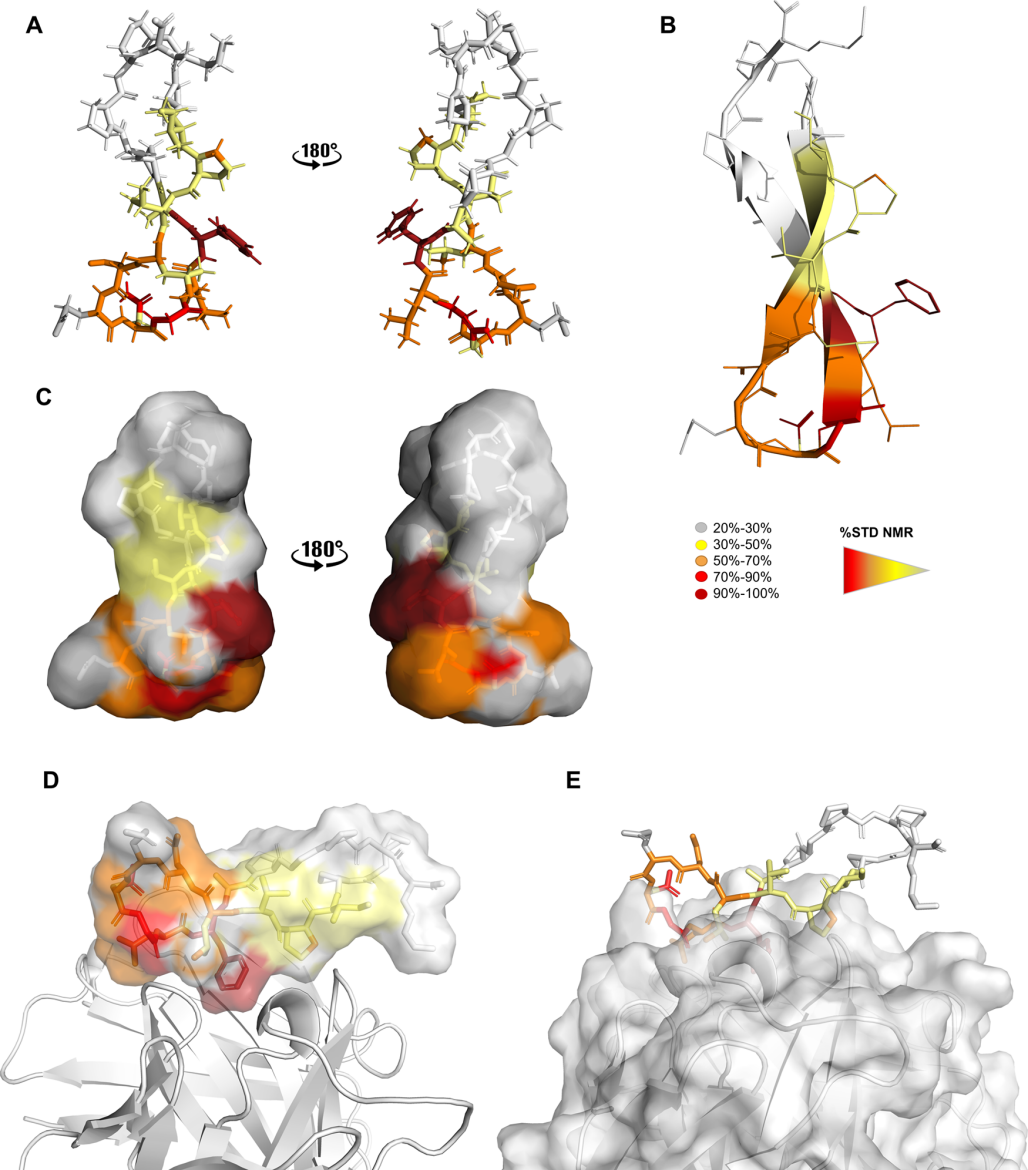
trNOESY-derived bioactive conformation of CP2 colored according to the STD effects (see legend). (A–C) Different renderings of CP2 in its bioactive conformation. (D,E) Different renderings of Fab 2C7–CP2 complex.

We determined the bioactive conformation of cyclic peptide CP2 when bound to 2C7 by complementary NOE-based experiments and MD simulations. We constructed a model of CP2 bound to Fab 2C7 using transferred nuclear Overhauser effects (trNOE) (Figure S8) experiments, where a sufficient off-rate of the peptide from the antibody allowed us to derive interproton distances from the bound state of 2C7 (Table S5). The spectrum exhibited a substantial number of trNOEs, including high- and medium-volume signals, suggesting the presence of a secondary structure and a well-defined structure in the presence of the Fab. This result agreed with those from ITC measurements, which exhibited favorable enthalpic and entropic terms. A summary of sequential NOE connectivities of the peptide in the bound state is shown (Table S5). Two hundred distance restraints were used to generate an ensemble of 2C7 structures with the program CYANA, which were further refined using AMBER18 (see Methods). We modeled the bioactive conformation of CP2 in the mAb 2C7 binding site, which we obtained from the crystal structure of Fab 2C7 (see above), and performed MD simulations (Figures S9–S11). A representative CP2 conformer obtained from MD cluster analysis colored according to the interacting epitope derived from STD binding studies is shown in Figure 5.

**Figure 6:**
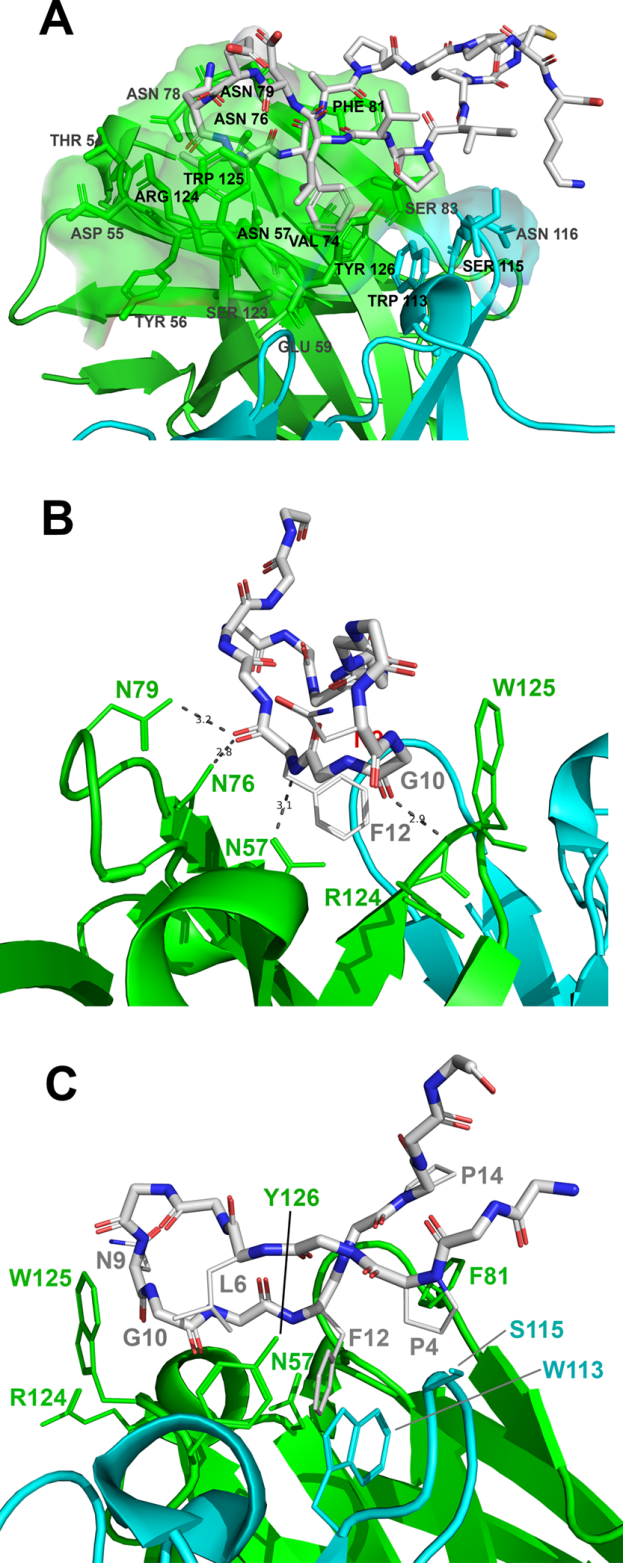
Interacting amino acid residues of the Fab 2C7–CP2 peptide complex. (A) Interacting residues observed in the MD minimized structure. The Fab heavy chain is shown in green, the light chain is shown in aqua, and CP2 is shown in stick representation colored by atom. (B) H-bond interactions between the Fab heavy chain and peptide as seen in the crystal structure. The coloring is same as in panel A. (C) Residues involved in hydrophobic interactions between Fab and peptide. The interacting residues from the crystal and MD minimized structures are given in Table 2.

The most representative frames for each cluster showed similar interactions between the peptide and the combining site (Figure S9). As shown by the backbone superposition of the most populated structures resulting from the MD cluster analysis (Figure S9C), a good convergence was observed. The NOE-based conformation of CP2 was retained in the 2C7 binding site during MD simulation, and the contacts found at the interface of the most populated conformers matched those from the X-ray data (Table 2 and Figure S10), further confirming the STD-derived binding epitope. The three-dimensional (3D) structure shown in Figure S10 illustrates the basic structural features of CP2 as well as the binding epitope. The residues forming the binding interface confirmed that mAb 2C7 primarily recognized CP2 residues Asn9, Gly10, Leu11, and Phe12 through polar and hydrophobic interactions (Table 2 and Figure S5). In particular, residue Phe12 of CP2 made H-bonds with the side chains of Asn57, Asn76, and Asn79 of the heavy chain. In addition, the backbone oxygen atoms of CP2 residues Asn9 and Gly10 formed H-bonds with Arg124 and Trp125 of the Fab heavy chain, respectively (Figure 6). CP2 peptide residue Asn9 formed a hydrophobic amide–pi interaction with Trp125 of the Fab heavy chain, and CP2 residue Leu11 mediated hydrophobic interactions with Asn57 and Arg124 of the heavy chain. CP2 residue Pro4 formed hydrophobic interactions with Ser115 (light chain) and pi-alkyl with Phe81 (heavy chain) and Trp113 (light chain). Leu6 and Pro14 mediated pi–alkyl interactions with Tyr126 and Phe81 (heavy chain), respectively (Table 2 and Figure 6). The carboxyl-terminus of the peptide, including Pro16, Cys17, and Lys18, were more solvent-exposed and more dynamic along the MD simulation. Overall, binding and MD studies of CP2 with Fab 2C7 confirmed the binding epitope and the bioactive conformation as described above and the proposed model of the Fab 2C7–CP2 complex (Figures 5, 6, and S9–S11).

**Table 2:** Polar Contacts between Peptide and Fab before and after MD Simulation

peptide residue	Fab residue^a^			
	X-ray contacts	type	MD contacts	type
Pro 4	Phe 81	pi-alkyl	Phe 81	pi-alkyl
	Ser 115^LC^	carbon	Ser 115^LC^	carbon
	Trp 113^LC^	pi-alkyl	Trp 113^LC^	pi-alkyl
Leu 6	Tyr 126	pi-alkyl	Tyr 126	pi-alkyl
	Trp 125	pi-alkyl	–b	–
Asn 9	Trp 125	amide pi-stacked	Trp 125	amide pi-stacked
	–	–	Arg 124	H-bond
Gly 10	Arg 124	carbon	Arg 124	carbon
	Trp 125	H-bond	Trp 125	H-bond
Leu 11	–	–	Arg 124	alkyl
	–	–	Asn 57	carbon
Phe 12	Trp 113^LC^	pi–pi stacked	–	–
	Val 74	pi-alkyl	Val 74	pi-alkyl
	Asn 57	H-bond	Asn 57	H-bond
	Asn 76	H-bond	Asn 76	H-bond
	Asn 79	H-bond	Asn 79	H-bond
Ala 13	Phe 81	amide pi-stacked	–	–
Pro 14	Phe 81	pi-alkyl	Phe 81	pi-alkyl

a Fab residue from heavy chain unless indicated by superscript LC.

b –, No contact, >3.5 Å.

## Discussion

In this study, we describe detailed structural and conformational analyses of the interaction between an antigonococcal LOS mAb and a cyclic mimetic peptide. We previously isolated a peptide, composed of 18 amino acid residues in which a disulfide bond linked Cys1 and 17.^19^ The 12-mer mimitope had three flanking amino acids at either end (CGP and GPC at the N- and C-terminus, respectively) derived from the thioredoxin protein in the pFLiTrx peptide display library.^19^ For our structural studies, we used variants of the peptide that were cyclized via a thioether bond, monomeric CP2, or tetrameric TMCP2 (Scheme S2), to circumvent the reduction of the disulfide bond present in the original peptide.^20^ We used a recombinant Fab fragment and the monomeric peptide CP2, which are the minimal structures needed to define the paratope and epitope to minimize conformational variability in crystallization experiments. We also used the Fab in SAXS studies because we expected that the peptide ligands would have larger proportional effects on the Fab compared with the mAb. These experiments permitted us to obtain information on the shape, conformation, and assembly state of the Fab fragment as it interacted with the TMCP2 tetramer. We conducted NMR-based experiments and native MS analyses on both the intact mAb and the Fab (Figures 1 and S12). MS experiments identified higher-order oligomers with the TMCP2 tetrapeptide; in these studies, we detected complexes comprising two mAbs or three Fabs, bound to a single tetrapeptide. These results suggested that steric hindrance prevents the binding of a third mAb or a fourth Fab to the CP2 tetramer. ITC studies further revealed that two CP2 molecules were accommodated by each mAb, leading to entropically and enthalpically favored interactions.

The crystal structure of the Fab 2C7-CP2 complex revealed that CP2 forms a class I beta-hairpin structure that interacts with the Fab predominantly through hydrophobic interactions. The hairpin is stabilized by hydrogen bonds between the two beta-strands of the hairpin and likely also by the thioether bond between the terminal residues. Peptide residues Pro4, Leu11, and Phe12 made the largest contributions to the buried surface area and solvation energy in the Fab-CP2 complex (Table S3). Of these, Leu11 and Phe12 were identified as consensus residues in the peptide display experiment that identified the original PEP1 sequence.^19^ Although Pro4 was not previously identified as a consensus residue, it mediated a pi-alkyl-stacking residue with Phe81 in the heavy chain of the Fab (Table 2). Peptide residue Asp7 formed a helix cap at the apex of the hairpin, and Gly10 was in a left-handed helical conformation. The role of these residues in the conformation of the peptide illuminates why these were consensus residues in the peptide display experiment to derive the mimitope.^19^

NMR analyses allowed us to map the binding epitope of the peptide, specifically, the amino acid residues establishing direct contact with the antibody. We performed MD simulations to model the 3D structure of the peptide bound to 2C7, which confirmed that the beta-hairpin conformation was involved in a stable interaction with 2C7, despite a certain flexibility of the solvent-exposed region at the covalently closed termini of the peptide. This is consistent with the fact that the central 12 amino acids (IPVLDENGLFAP) are unique to the peptide display library, while the flanking amino acids (CGP at the N-terminus and GPC at the C-terminus) are derived from the thioredoxin scaffold of the pFliTrx system used for panning to identify the mimitope.^19^

Interestingly, we compared the structure of the CP2 peptide in the crystal structure to that following MD simulations (Table 2, Figures S10 and S11). While an overall agreement was observed between X-ray and MD complexes, slight differences were revealed in the antibody–peptide molecular interactions. Specifically, there were three interactions present in the crystal structure but not in the most populated MD clusters, which included Leu6, Phe12, and Ala13 of CP2, all involving pi bonding interactions with Fab 2C7 (Table 2). There were also three interactions in the MD model but not in the X-ray structure, including an H-bond between CP2 residue Asn9 and heavy chain residue Arg124 and hydrophobic interactions with CP2 residue Leu11 (Table 2). Together, these differences suggest that the peptide is less constrained and more dynamic in solution. A more complete picture of the structure and dynamics of the CP2 peptide could be obtained through crystallographic studies of the peptide alone, which thus far have not been fruitful.

Antibody-bound peptides can adopt a broad range of conformations, often displaying a limited secondary structure. While a subset of complexes contained peptides classified as alpha helix or beta-hairpin motifs, most antibody-bound peptides were classified as a random coil or “other”.^33^ In addition, different antibodies can bind to the same peptide in various conformations.^33^ In our structure, the beta-hairpin conformation is promoted by peptide residue D7, which forms a helix cap, and Gly 10 at position + 3 adopts a left-handed helical conformation. It is noteworthy that the phage display consensus peptide sequence, DE_GLF, contains residues that are important for both binding and conformation.

Our structural information can be incorporated into a pharmacophore model for further development of optimized peptide vaccine candidates. As noted above, we did not observe electron density for the terminal residues of the CP2 peptide or its thioether linkage, which were exposed to solvent. We previously showed that circular dichroism spectra of the linear and cyclic versions of the peptide were similar,^19^ which may suggest the formation of a hairpin structure in the absence of the 2C7 antibody. Although linear versions of the mimitopes may also inhibit the binding of mAb 2C7 to gonococcal LOS,^19,20^ our data suggest that constraining the peptide to permit forming a beta-hairpin loop may maximize mAb 2C7 binding.

Finally, our structural data can be used to guide the humanization of mAb 2C7 for use as an adjunctive or preventive immunotherapeutic antibody. Identification of key residues that interact with CP2 could inform the design of humanized mAb variants with similar or higher affinity for the nominal LOS antigenic target. Conversely, CP2 could also be modified to increase binding to the 2C7 antibody, which might improve immunogenicity. Prior studies have suggested that MAPs composed of linear versions of the mimitope peptide are antigenic (i.e., can recognize mAb 2C7).^19,20^ The lack of contact of the amino acids that flank the central 12-mer peptide also suggests that this minimal structure alone when presented as a multimeric antigen may suffice for immunogenicity. As an example, a linear molecule devoid of flanking Cys residues would eliminate the formation of intra- and intermolecular disulfides that resulted in heterogeneity in a prior iteration of the octameric MAP antigen.^17^

## Methods

### Materials

For NMR studies, we used phosphate-buffered saline tablets (Sigma) dissolved in deionized water and 10% deuterium oxide 99.9 at % D (Sigma). For mass spectrometry (MS), grade water (W8-1) and acetonitrile (Thermo Scientific) were used. We obtained ammonium acetate (99.999% trace metal basis) from Merck-Sigma, and 99.9% formic acid ampules were from Pierce. For crystallization experiments, we employed sparse matrix screening reagents (Hampton Research) and molecular biology grade chemicals (Sigma, Fluka, and Hampton Research).

### Generation and Purification of Antibodies and Peptides

Chimeric mAb 2C7 and the sequence for the *V*_H_ and *V*_L_ regions of murine mAb 2C7 have been described previously.^18^ The gene sequence and the translated amino acid sequence used to generate Fab 2C7 are shown in Figure S13. Human-mouse chimeric mAb 2C7 was expressed in ExpiCHO cells and purified by affinity chromatography of tissue culture supernatants using Protein A/G agarose. Chimeric Fab 2C7 was expressed in CHO cells and purified by affinity chromatography with an anti-CH1 antibody (LakePharma).

CP2 and TMCP2 were synthesized by AmbioPharm, Inc., as described previously.^20^ Briefly, the thioether monomer was synthesized on a Fmoc-Lys-Wang resin using standard Fmoc-tBu amino acid derivatives. At the completion of assembly of the protected linear peptide sequence: H-Gly-Pro-(Ile-Pro-Val-Leu-Asp-Glu-Asn-Gly-Leu-Phe-Ala-Pro)-Gly-Pro-Cys-Lys-OH, bromoacetic acid was coupled to the amino terminus via symmetric anhydride coupling. The peptide Br-Ac-Gly-Pro-Ile-Pro-Val-Leu-Asp-Glu-Asn-Gly-Leu-Phe-Ala-Pro-Gly-Pro-Cys-Lys-OH was cleaved from the solid support and simultaneously deprotected using trifluoroacetic acid containing H_2_O and triisopropyl silane as cationic scavengers. The crude linear peptide was purified using preparative HPLC and subsequently cyclized by dilution into a 1% Na_2_CO_3_ buffer to a concentration of 0.3 mg/ml. Cyclization was completed at 18 h, and the cyclic thioether peptide was purified via preparative RP-HPLC. Fractions that had a purity of > 90% by analytical HPLC were pooled and lyophilized. The cyclic thioether monomer peptide was characterized using ESI-MS (calculated *m*/*z* = 1864.17; measured *m*/*z* = 1864.88). Linking of the CP2 monomers to a tetra-MAP core to yield TMCP2 has been described previously.^20^

### Size Exclusion Chromatography-Ultraviolet–Mass Spectrometry (SEC-UV-MS) Analysis

We prepared solutions of mAb and Fab 2C7 at a concentration of 35 μM in MS-grade water. To this solution, TMCP2 peptide diluted in MS-grade water was added in a ratio of 1:4 (mol/mol). Twenty-five micrograms of protein samples were analyzed via SEC-UV-MS, either pure or mixed with TMCP2. For the SEC separation, an ACQUITY UPLC BEH200 SEC, 4.6 × 150 mm, 1.7 μm column (Waters) was employed. A Vanquish Flex UHPLC column (Thermo Scientific) was used, employing 50 mM ammonium acetate as the mobile phase and delivering an isocratic gradient with a flow of 0.3 mL/min, while the column temperature was kept at 30 °C. UV analysis was performed using a diode array detector set at a wavelength of 280 nm. uHPLC was connected to a Q Exactive UHMR hybrid quadrupole Orbitrap mass spectrometer (Thermo Scientific) equipped with an IonMax source with an HESI-II probe and a high-flow, 32-gauge needle. Full MS spectra were acquired in positive polarity in a scan range of 1000–15,000 *m*/*z*. The resolution was set to 6250 at *m*/*z* 400, with an acquisition gain control (AGC) target of 1 × 10^6^ ions, and 10 microscans were performed. The maximum injection time was 200 ms. In-source trapping desolvation was set to −50 V, and the trapping gas pressure was set to 7.0. Detector *m*/*z* optimization was set to low *m*/*z*, while the ion transfer target *m*/*z* was set to high. Sheath gas was set to 30 arbitrary units (AU) and auxiliary gas was set to 15 AU. The spray voltage was 3.8 kV, the capillary temperature was 320 °C, the S-lens RF was set to 200 V, and the auxiliary gas heater temperature was 250 °C.

#### Reversed Phase-Mass Spectroscopy

TMCP2 sample was analyzed through reversed phase chromatography using a mass spectrometer as the detector (RP-MS). The analysis was carried out on a Vanquish Flex UHPLC equipped with a MAbPac RP 2.1 × 50 mm column (Thermo Scientific) performing a linear gradient from 20% B to 40% B in 8 min, where mobile phase A was 0.1% formic acid in MS-grade water and B was 0.1% formic acid in MS-grade acetonitrile. The column temperature was kept at 80 °C. After LC separation, the sample was introduced into an Orbitrap Exploris MX mass detector (Thermo Scientific). The spray voltage was 3.8 kV, and the sheath gas and auxiliary gas were set to 25 and 10 AU, respectively. Ion transfer tune temperature and vaporizer temperature were set to 320 and 150 °C, respectively. The selected application mode was “Peptide,” while the pressure mode was standard. Scan settings were as follows: Orbitrap resolution of 120,000 (at 200 *m*/*z*), scan range between 600 and 3000 *m*/*z*, AGC was 300%, maximum injection time was 200 ms, and RF lens % was 80.

#### MS Data Processing

We processed raw data for all MS experiments using BioPharma Finder version 4.1 (Thermo Scientific). For analysis of TMCP2, the Xtract algorithm was employed, selecting the average over the selected time range option. TIC was deconvoluted between 5.3 and 5.6 min. The output mass range was set between 1000 and 10,000 Da, the charge range between 1 and 10, and the minimum adjacent charges number was set to 2. For mAb and Fab deconvolution, the ReSpect algorithm was used, selecting the Sliding Window option. The TIC for mAb analysis was deconvoluted between 2.5 and 8 min using a target average spectrum width of 0.3 min and a scan offset of 1. Output and model mass range were between 7000 and 500,000 Da, and charge states deconvolution mass tolerance was set to 20 ppm. Charge states ranged from 5 to 60 and a minimum number of adjacent charges was set to 3. For Fab analysis, similar parameters were used, but the mass range was 5000–200,000 Da.

### NMR Spectroscopy

NMR spectra were recorded on a Bruker AVANCE NEO 600-MHz equipped with a cryo probe, and data acquisition and processing were performed with TOPSPIN 4.1.1 software. Samples were prepared in H_2_O:D_2_O (90:10) buffered with 50 mM phosphate at pH 7.4, using 3 mm NMR tubes. NMR spectra were acquired using antibody concentrations of 10–20 μM, a peptide concentration of 1 mM, and an antibody:peptide ratio from 1:18 to 1:50, depending on the NMR experiments, and temperatures of 288 and 298 K; 0.05 mM amount of deuterated trimethylsilylpropanoic acid (TSP) was used as an internal standard.

#### Peptide Assignment

Assignment of the resonances of the CP2 peptide in complex with Fab 2C7 (10:1) was obtained by the analysis of the COSY, TOCSY, and NOESY spectra using TOPSPIN and CARA.^34,35^

#### trNOESY Analysis

Homonuclear 2D 1H-1H NOESY experiments were carried out by using datasets of 4096 × 900 points and mixing times of 100–300 ms. The interproton cross-relaxation rates (σ*_ij_*) were measured by the integration of the NOE cross peaks of interest. The experimental distances (*r_ij_*) were then obtained by the automatic calibration protocol in CYANA.

#### STD NMR Analysis

STD NMR experiments were acquired with 32,000 data points and zero-filled up to 64,000 data points prior to processing. The protein resonances were selectively irradiated using 40 Gauss pulses with a length of 50 ms, setting the off-resonance pulse frequency at 40 ppm and the on-resonance pulse at 5.5 ppm. An excitation sculpting with gradient pulses (esgp) was applied for the suppression of water signals. The %STD displayed in the epitope map of the ligand was obtained by the ratio of the STD signals in the STD spectrum (*I*_0_ – *I*_sat_) and each relative peak intensity of the unsaturated reference spectrum (off-resonance, *I*_0_), at saturation time of 2 s. The highest STD signal was set to 100% and all the other STD values were normalized to this value. We measured the *K*_D_ by titrating an increasing concentration of CP2 (0.17, 0.24, 0.30, 0.37, 0.55, and 1.70 mM) into a solution of mAb. Ligand was titrated into the protein sample from concentrated stock solutions to minimize dilution effects. The one-site binding model has been used, and a homogeneous antibody binding was assumed. STD-AF values, at a saturation time of 2 s, were plotted as a function of the concentration of the ligand in order to construct the binding (Langmuir) isotherm.

#### WaterLOGSY Analysis

The waterLOGSY experiments were performed on CP2 in the absence and presence of the mAb, using a conventional 1D NOE-ePHOGSY pulse sequence^36^ provided in the Bruker library (ephogsygpno.2). The bulk water was selectively irradiated by 1D NOESY, and the solvent suppression was modified into a double pulsed-field gradient (DPFG) perfect-echo.^37^ A mixing time of 2s was used in order to compare the WaterLOGSY experiment with the STD NMR experiment.

#### CPMG Analysis

A mixture of 20 μM mAb2C7 and 1 mM CP2 peptide was prepared in deuterated phosphate saline buffer pH 7.4. Pseudo 2D experiments (cpmg_esgp2d) were used for detecting T2 spin–spin relaxation measurements. Data were analyzed by fitting the equation *f*(*t*) = *I*_o_ × exp (−*t*/*T*) using Dynamics Center 2.7.1.

#### Diffusion Ordered Spectroscopy Analysis

Pulsed-field gradient (PFG)-NMR experiments were performed on TCMP2 in the absence and presence of mAb2C7 to calculate the diffusion coefficients of the molecules by using an antibody:tetrapeptide molar ratio of 1:5. Data were analyzed through Dynamics Center 2.7.1 by using the following decay function: *I* = *I*_0_ exp (−D*(G*γ*δ)2*(Δ – δ/3 – τ/2)), where *I* is the intensity of the integrated proton signal, *I*_0_ is the reference signal when magnetic field gradient is not applied, *D* is the diffusion coefficient, γ stands for the gyromagnetic ratio, *G* represents the gradient strength, and Δ and δ are the parameters that define the diffusion time (big delta) and diffusion gradient length (little delta). 2D diffusion ordered spectroscopy NMR experiments were set with a linear gradient incremented, in 64 steps, from 2 to 95%, using Δ = 200 ms and δ = 3 ms.

### Small-Angle X-ray Scattering

SAXS experiments were conducted to study the solution behavior of Fab 2C7 alone and upon its interaction with TMCP2 using a 20 μM Fab concentration and a Fab:TMCP2 molar ratio of 1:5. These experiments were performed at Diamond Light Source beamline B21 (Didcot, U.K.); the experimental SAXS curves are shown in Figure 1C. From the experimental data, it is possible to calculate the *P*(*r*) function through the indirect Fourier transformation^38^:
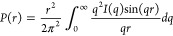
1where *P*(*r*) represents the atomic distribution in space, which is used to obtain useful information about the scattering particle such as the shape in real space.

B21 beamline configuration consisted of a beam energy of 12.4 keV, and a sample-to-detector distance of 3.7 m. This setup allowed us to collect the data for the scattering vector modulus *Q* = 4πsin(θ/2)λ in the range of values between 0.0045 and 0.34 Å^–1^, where θ is the scattering angle.^39^ SAXS data reduction was performed with SCATTER IV software. We used the ATSAS program package version 3.1.3^40^ for data analysis. Pair correlation function, *P*(*r*), calculations were carried out using GNOM software.^41^ We performed ab initio shape reconstructions with the DAMMIF program^42^ employing dummy residues and constraints provided by the SAXS profile. For each data set, we generated a total of 20 reconstructions and averaged them, using DAMAVER software,^43^ to obtain the most representative final model for each sample and finally refined with DAMMIN software.^44^

### Isothermal Titration Calorimetry

Isothermal titration calorimetry (ITC) experiments were carried out at 20 °C by using a MicroCal PEAQ-ITC instrument. mAb and peptide CP2 were both dialyzed against PBS pH 7.4 to avoid buffer mismatch between solutions. A solution of mAb with a concentration of 30 μM was loaded into the cell, and for each measurement, CP2 was added to the antibody solution through a microsyringe under a stirrer speed of 750 rpm. The titration was performed by adding 2 μL from a 1.67 mM CP2 solution for 19 injections using a time duration of 150 s. The data were analyzed and fitted by using the one-binding site model. The standard thermodynamic parameters of equilibrium were calculated by the following equation^45^:
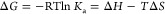
2where Δ*G* is the Gibb’s free energy, *K*_a_ is the equilibrium constant, *R* is the gas constant, *T* is the temperature in kelvin, Δ*H* is the enthalpy change, and Δ*S* is the entropy change.

### Crystallization, X-ray Data Collection, and Analysis

Crystals of Fab 2C7 grew in sitting drops from an equal mixture of 8 mg/ml Fab 2C7 and a precipitant solution containing 0.085 M sodium HEPES, pH 7.5, 8.5% (v/v) 2-propanol, 17% (w/v) PEG 4000, and 15% (v/v) glycerol (Hampton Research). We collected single wavelength (λ = 1.00 Å) X-ray data on beamline 8.3.1 at the Advanced Light Source, Lawrence Berkeley National Laboratory. We used the sequence of chimeric Fab 2C7 to generate a search model using Swiss-Model (https://swissmodel.expasy.org),^46^ which identified human-mouse chimeric Fab (PDB ID 6RCO)^47^ as a top-scoring candidate. We solved the structure by MR using Phenix-MR;^48^ the crystals were in space group P1 with two copies of the Fab in the asymmetric unit. We refined the structure without noncrystallographic symmetry restraints using iterative refinement with Phenix.refine^49^ and manual model building with Coot^50^ until the R-free converged. Bond lengths, angles, and other geometric statistics were calculated with MolProbity^51^ in the later rounds of refinement.

We formed the Fab-peptide complex by incubating a 1:5 molar ratio of Fab to peptide at 4 °C for 30 min. We concentrated the complex by centrifugal filtration (Spin-X UF6, 10K MWCO, Corning). We used UV spectroscopy (NanoDrop 1000, ThermoScientific) to measure the protein concentration and calculated the extinction coefficient of the concatenated Fab heavy and light and peptide sequences (ProtParam^52^). We grew crystals of the Fab 2C7-CP2 complex in hanging drops from an equal mixture of 8 mg/ml complex and a precipitant solution containing 0.095 M trisodium citrate, pH 5.6, 20% (v/v) 2-propanol, 19% (w/v) PEG 4000, and 5% glycerol (Hampton Research). We collected X-ray data as above and solved the structure by MR with Phenix-MR^48^ using the structure of Fab 2C7 alone as a search model. The crystals grew in space group P2_1_ with two copies of the complex in the asymmetric unit. We refined the structure using software and methods described for Fab 2C7 above. The data collection and refinement statistics for both structures are given in Table S1.

We analyzed the crystallographic data with PyMol (PyMOL Molecular Graphics System, version 2.0 Schrödinger, LLC.) and PISA.^32^ The coordinates are available in the Protein Data Bank (http://www.rcsb.org) with accession codes 8DOZ for Fab 2C7 alone and 8DUZ for the Fab 2C7-CP2 complex. Figures derived from crystal structures and models from MD simulations were prepared using PyMol (The PyMOL Molecular Graphics System, Version 2.0 Schrödinger, LLC.).

### Structure Calculation and Refinement; MD Simulations

Proton–proton distance restraints were derived from the analysis of the 2D NOESY spectrum acquired as described earlier. A total of 200 distance restraints were obtained from this analysis and used for structure calculation. The program CYANA 2.1^53^ was used to calculate a family of 600 structures of the cyclic peptide starting from randomly generated conformers in 15,000 annealing steps. Upper and lower distance limits as well as a linkage were imposed between the sulfur atom of Cys17 and the carbon of the methylene group to form the thioether bond that closes the cyclic structure of the peptide. The restraints and the statistics of the obtained NMR structures are reported in Table S5. The quality of the structures calculated by CYANA was assessed by a properly defined energy function (target function) proportional to the squared deviations of the calculated restraints from the experimental ones plus the standard covalent and nonbonded energy terms. The 30 structures with the lowest target function were analyzed. Structure refinement and MD simulations were performed with AMBER as described later.

#### MD Simulations

The CP2 peptide in its NMR-derived bioactive conformation (see above) was modeled into the Fab crystal structure binding pocket and then further refined using MD simulations with AMBER18.^54^ The complex was refined by preprocessing the structure using Maestro Protein Preparation Wizard (Maestro, version 9.2. Schrodinger, LLC). Prior to the MD simulation, the complex was minimized using Sander in Amber tools. Charges of the proteins and atom types were determined by using the AMBER ff14SB force field. In the Leap module, Na^+^ ions were added to neutralize the complex and an octahedral box containing explicit TIP3P water molecules buffered at 10 Å was chosen to hydrate the molecular structure. MD simulations were run using the pmemd.cuda implementation within the AMBER18 package. Long-range electrostatic interactions were calculated using Ewald’s smooth particle mesh method, with each simulation performed under periodic boundary conditions, and the grid spacing was fixed at 1 Å. During equilibration, the system was minimized by applying a restriction to the protein which was gradually removed in the following steps: (1) a slow thermalization of the system was carried out from 0 to 300 K applying a solute restraint; (2) the temperature was increased from 0 to 100 K at constant volume; (3) then, from 100 to 300 K in an isobaric ensemble and kept constant at 300 K for 50 ps with progressive energy minimization and solute restraint; (4) once the process was completed, the restraints were removed and the systems then continued in an isothermal–isobaric ensemble along the production. Coordinates were collected as 10,000 structures along MD. Trajectories were analyzed using the ptraj module within AMBER18 and visualized using VMD.^55^ A cluster analysis with respect to the ligand RMSD using the K-mean algorithm implemented in the ptraj module was calculated on the trajectories. The representative pose of the most populated cluster was considered a model to depict the interactions of the complex.

Hydrogen bonds were calculated using the CPPTAJ module in AMBER18 and defined as occurring between an acceptor heavy atom A, a donor hydrogen atom H, and a donor heavy atom D. The A-H-D angle cutoff was 135° with a distance cutoff set to 3 Å. The frequency of the antibody-peptide bonds established during the dynamics is reported with a cutoff of 5 Å.

## Supporting Information Available

Five tables describing crystal and NMR structural details; two schemes showing LOS, cyclic peptide 2 (CP2), and tetra-MAP CP2 (TMCP2); thirteen figures; additional details of SEC, NMR, SAXS, ITC, and X-ray crystallography experiments and MD simulations; and last figure shows the sequence of Fab 2C7 used in structural studies (PDF)
