# Supplementary material for: Gonococcal Mimitope Vaccine Candidate Forms a Beta-Hairpin Turn and Binds Hydrophobically to a Therapeutic Monoclonal Antibody
Source: JACS Au. 2024 Jul 5;4(7):2617–29. doi: 10.1021/jacsau.4c00359 (PMC11267536; doi:10.1021/jacsau.4c00359)
Supplement: Supplementary file 1 — au4c00359_si_001.pdf [file au4c00359_si_001.pdf]

Supporting Information

Gonococcal mimitope vaccine candidate forms a beta-hairpin turn and binds hydrophobically to a therapeutic monoclonal antibody

Peter T. Beernink[1#*], Cristina Di Carluccio[2#], Roberta Marchetti[2#], Linda Cerofolini[3], Sara Carillo[4], Alessandro Cangiano[2], Nathan Cowieson[5], Jonathan Bones[4,6], Antonio Molinaro[2], Luigi Paduano[2], Marco Fragai[3], Benjamin P. Beernink[1], Sunita Gulati[7], Jutamas Shaughnessy[7], Peter A. Rice[7], Sanjay Ram[7] and Alba Silipo[2*]

[1]Department of Pediatrics, University of California, San Francisco, 5700 Martin Luther King Jr. Way, Oakland, California, 94609 USA;

[2]Department of Chemical Sciences, University of Naples Federico II, Via Cintia 4, 80126 Naples, Italy;

[3]Department of Chemistry, University of Florence, Via della Lastruccia 13, 50019 Sesto Fiorentino, Italy

[4]National Institute for Bioprocessing Research and Training, Foster Avenue, Mount Merrion, Blackrock, Co., Dublin, Ireland. A94 X099;

[5]Diamond Light Source, OX11 0DE Didcot, Oxfordshire, England, UK

[6]School of Chemical and Bioprocess Engineering, University College Dublin, Belfield, Dublin 4, Ireland;

[7]Department of Infectious Diseases and Immunology, University of Massachusetts Chan Medical School, 364 Plantation St., Worcester MA 01605 USA

#Joint first authors
*To whom correspondence should be addressed:
Peter T. Beernink: peter.beernink@ucsf.edu
Alba Silipo: silipo@unina.it

Supporting Tables

Table S1. Data collection and refinement statistics[a]

	Fab 2C7 (PDB ID 8DOZ)	Fab 2C7-CP2 complex (PDB ID 8DUZ)
Wavelength	1.00	1.00
Resolution range (Å)	52.97 – 1.70	58.19 – 1.65
Space group	P1	$P2_1$
Unit cell		
a, b c (Å)	40.00, 50.61, 106.2	58.61, 64.19, 110.81
α, β, γ (°)	92.13, 86.78, 82.26	90.00, 97.12, 90.00
No. total reflections	294,917 (26,592)	660,791 (64,843)
No. unique reflections	82,948 (7,601)	97,770 (9,710)
Multiplicity	3.6 (3.5)	6.8 (6.7)
Completeness (%)	91.6 (83.8)	99.5 (99.4)
Mean I/sigma(I)	19.0 (2.1)	11.1 (1.0)
Wilson B-factor ($Å^2$)	20.3	19.7
R_{merge}	0.044 (0.859)	0.087 (1.841)
R_{meas}	0.052 (0.997)	0.094 (1.997)
R_{pim}	0.027 (0.504)	0.036 (0.766)
$CC_{1/2}$	0.999 (0.789)	0.999 (0.461)
No. reflections used in refinement	80,960	95,844
No. reflections used for R-free	1,325	1,894
R-work	0.177	0.189
R-free	0.216	0.234
No. non-hydrogen atoms		
macromolecules	6,346	6,566
ligands	19	26
solvent	538	580
No. protein residues	839	865
RMS bond lengths (Å)	0.010	0.010
RMS angles (°)	1.139	1.065
Ramachandran favored (%)	96.97	96.23
Ramachandran allowed (%)	3.03	3.78
Ramachandran outliers (%)	0.00	0.00
Rotamer outliers (%)	0.42	0.67
Clashscore	1.04	1.92
Average B-factor		
macromolecules	31.70	32.9
ligands	49.73	52.82
solvent	31.35	40.0

[a] statistics for the highest-resolution shell shown in parentheses

Table S2. Buried surface area and solvation energy between Fab 2C7 and peptide CP2 chains[a]

Chain 1 (Fab)	Chain 2 (CP2)	Buried Surface Area vs. CP2 (Å^2)	Solvation energy vs. CP2 (D^iG)	No. H-bonds with CP2
A (heavy)	F	482.0	-7.6	4
B (light)	F	95.1	-2.2	0
C (heavy)	E	469.7	-7.1	4
D (light)	E	86.9	-2.1	0

[a] calculated with Protein Interactions, Surfaces and Assemblies (PISA) server (https://www.ebi.ac.uk/msd-srv/prot_int/pistart.html) (E. Krissinel and K. Henrick (2007) J. Mol. Biol. 372, 774-797)

Table S3. Analysis of residues interacting between Fab 2C7 and peptide CP2[a]

Chain 1 (Chain ID)	Chain 1 Residue	Chain 2 (Chain ID)	Buried Surface Area (Å^2)[b]	Solvation energy (D^iG)[b]	H-bond
Heavy (A/C)	Asn57	CP2 (F/E)	39.7 / 41.4	-0.48 / -0.48	Yes
Heavy (A/C)	Asn76	CP2 (F/E)	35.4 / 32.9	-0.28 / -0.31	Yes
Heavy (A/C)	Asn79	CP2 (F/E)	27.3 / 23.1	-0.30 / -0.26	Yes
Heavy (A/C)	Phe81	CP2 (F/E)	102.8 / 86.2	1.64 / 1.38	No
Heavy (A/C)	Trp125	CP2 (F/E)	86.9 / 86.0	0.93 / 0.93	Yes
Heavy (A/C)	Tyr126	CP2 (F/E)	77.3 / 72.8	0.51 / 0.51	No
Light (B/D)	Trp113	CP2 (F/E)	36.0 / 35.7	0.56 / 0.57	No
CP2 (F/E)	Pro4	Heavy (A/C)	44.6 / 36.8	0.55 / 0.50	No
CP2 (F/E)	Leu6	Heavy (A/C)	65.3 / 67.0	1.03 / 1.04	No
CP2 (F/E)	Asn9	Heavy (A/C)	29.7 / 34.8	-0.02 / 0.00	No
CP2 (F/E)	Gly10	Heavy (A/C)	40.4 / 38.5	0.14 / 0.09	Yes
CP2 (F/E)	Leu11	Heavy (A/C)	107.9 / 111.1	1.73 / 1.78	No
CP2 (F/E)	Phe12	Heavy (A/C)	129.0 / 129.2	1.34 / 1.36	Yes
CP2 (F/E)	Pro14	Heavy (A/C)	31.4 / 33.7	0.49 / 0.53	No
CP2 (F/E)	Pro4	Light (B/D)	48.7 / 54.2	0.69 / 0.79	No
CP2 (F/E)	Phe12	Light (B/D)	34.0 / 35.5	0.54 / 0.57	No

[a]interactions identical in both copies

[b]the two values refer to first chain/second chain (i.e. A/C, B/D, or F/E)

[a] calculated with Protein Interactions, Surfaces and Assemblies (PISA) server (https://www.ebi.ac.uk/msd-srv/prot_int/pistart.html) (E. Krissinel and K. Henrick (2007) J. Mol. Biol. 372, 774-797)

Table S4. Water molecules that bridge interactions between Fab 2C7 and peptide CP2

CP2 chain / atom	Water No.	Fab atom	D1 / D2 (Å)[a]
F / Pro2 O	665	B / Asn116 ND2	3.0 / 3.3
F / Pro4 O	435	B / Trp113 NE1	3.6 / 2.8
F / Pro4 O	435	A / Tyr126 OH	3.6 / 2.7
F / Leu6 N	270	B / Trp113 NE1	3.7 / 2.6
F / Asp7 OD2[b]	74	A / Asn76 ND2	2.7 / 3.0
F / Asn9 O	189	A / Asp55 O	2.8 / 2.7
F / Gly10 O	258	A / Asn57 ND2	2.7 / 2.9
E / Pro4 O	708	C / Phe126 OH	3.0 / 3.2
E / Pro4 O	708	D / Ser115 O	3.0 / 2.7
E / Asp7 OD2	388	C / Asn76 ND2	2.7 / 3.0
E / Asn9 O	562	C / Arg124 NH2	2.6 / 3.1
E / Gly10 O	561	C / Asn57 ND2	2.8 / 2.9
E / Phe12 N	561	C / Asn57 ND2	3.6 / 2.9

[a] interactions identical in both copies

[b] the two values refer to first chain/second chain (i.e., A/C, B/D, or F/E)

Table S5. Restraints used in the structure calculation of CP2

NOE Restraints	Number
Intraresidue (i=j)	40
Interresidue	160
Sequential (|i–j| = 1)	72
Medium range (1 < |i–j| < 4)	47
Long range (|i–j| > 5)	41
Total	200
Average RMSD from the mean (Å)	
Backbone	0.6 ± 0.18
Heavy atoms	1.3 ± 0.27
Residual CYANA target function	0.9 ± 0.03

Supporting Figures

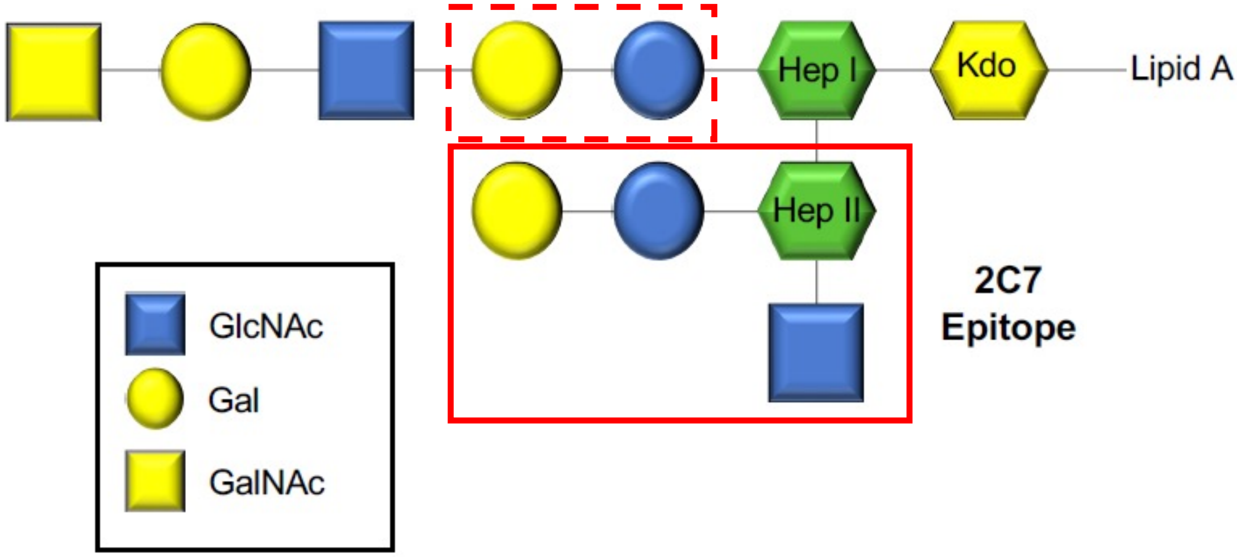

Scheme S1. *Neisseria gonorrhoeae* LOS glycan structure. Based on data from Gulati et al. (J Infect Dis. 1996;174(6):1238-48) and Yamasaki et al. (J Biol Chem. 1999;274(51):36550-8), the glycan structure recognized by mAb 2C7 is indicated in the solid red box. A role for lactose from HepI (dashed red box) could not be excluded.

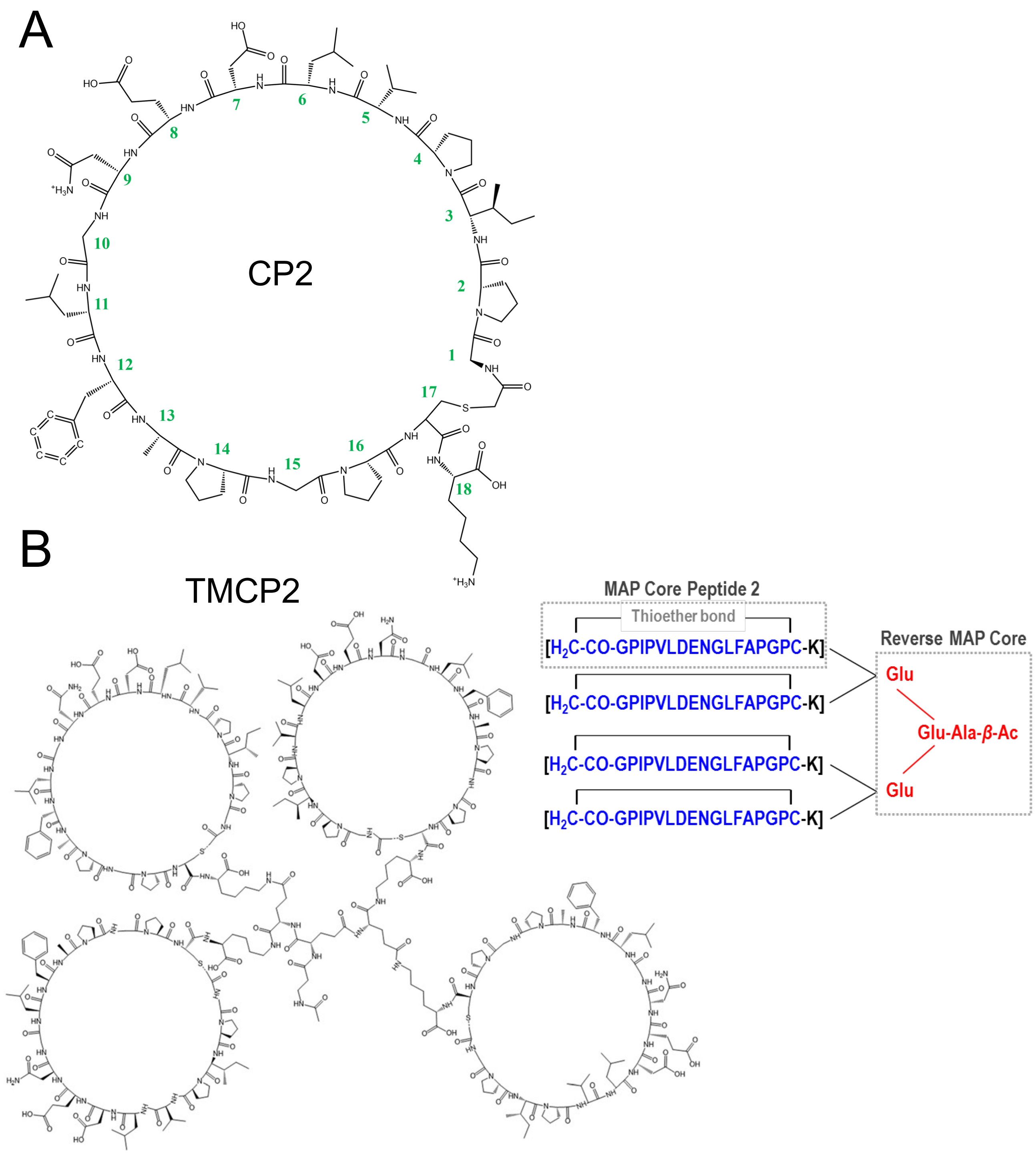

Scheme S2. **A,** Monomeric cyclic peptide 2 (CP2) cyclized via a thioether bond. **B,** Tetrameric multi-antigen peptide (MAP) CP2 (TMCP2). Chemical structure shown on the left and amino acid sequence shown on the right. Chemical formula (free base): $C_{360}H_{542}N_{84}O_{108}S_4$, average molecular weight (free base): 7903.02 Da.,monoisotopic mass: 7897.84.

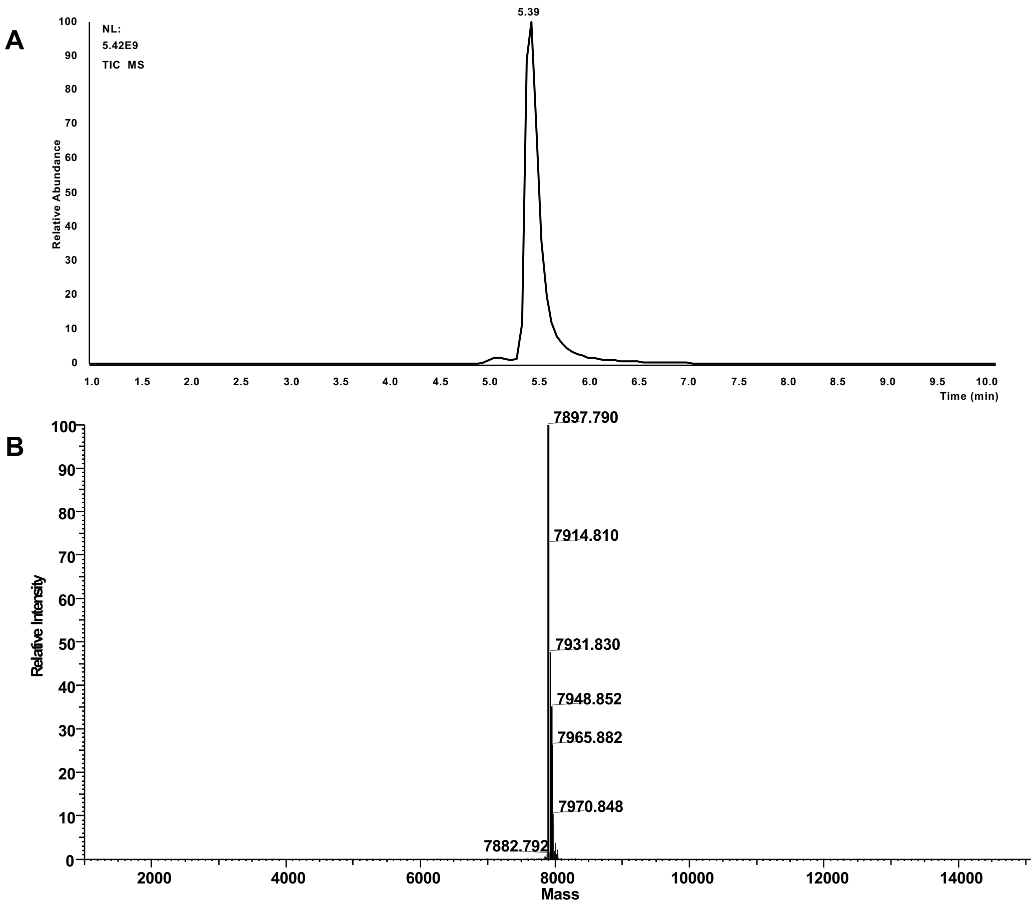

Figure S1. Reversed phase-MS analysis of TMCP2. **A,** Total ion chromatogram (TIC) of the RP-MS analysis of TMCP2. TIC showed a main species eluting at 5.39 minutes which, upon deconvolution, returned the spectrum depicted in panel B (99.5 % purity based on integration of TIC trace). **B,** Monoisotopic mass for TMCP2 was 7897.790 Da and average mass was 7902.81 Da.

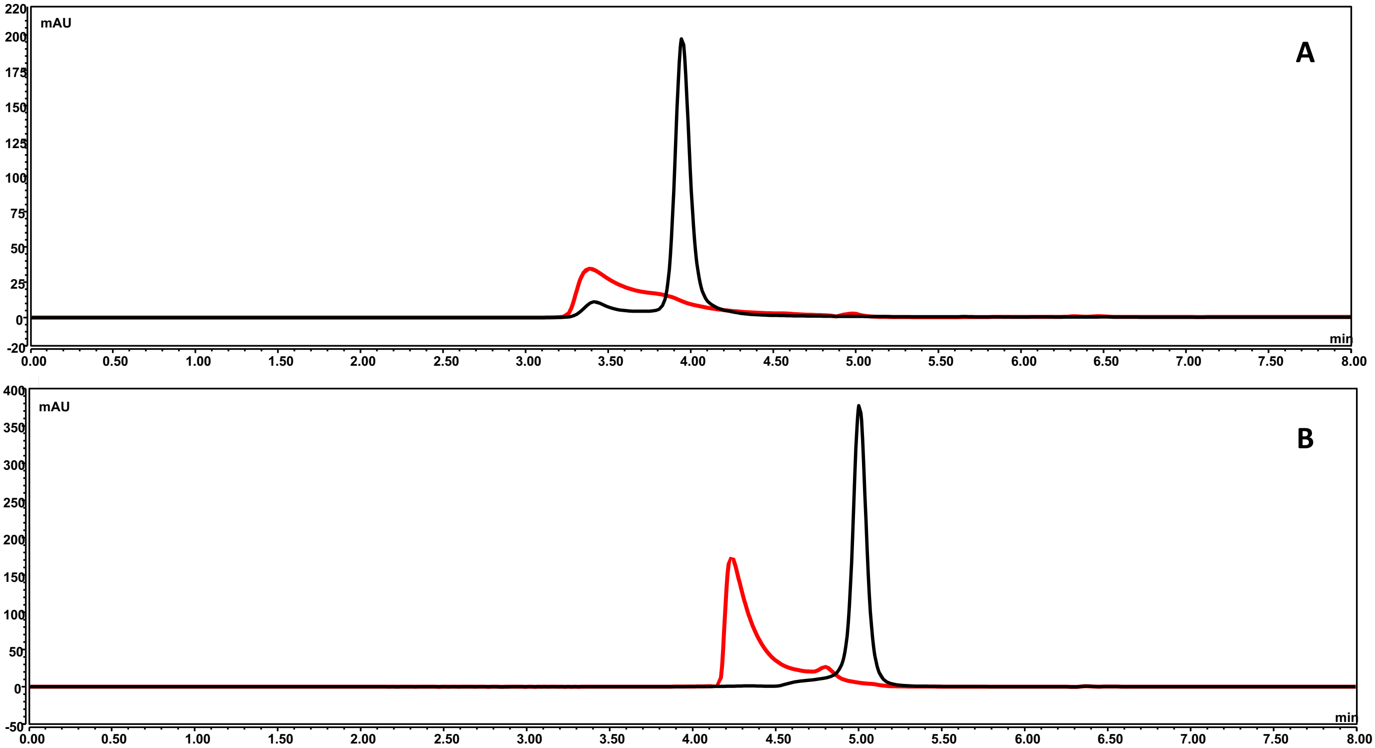

Figure S2. SEC-UV analysis of mAb and Fab complexes with TMCP2 tetramer. **A,** Overlay of the SEC-UV chromatograms of: mAb 2C7 (black trace) and mAb:TMCP2 (1:4 molar ratio, red trace). **B,** Overlay of Fab 2C7 (black trace) and Fab 2C7:TMCP2 complex (1:4 molar ratio, red trace). The tailing of SEC profiles could be explained by multiple species, as the interaction of either the mAb or Fab could form with any of the four peptide subunits of tetrameric TMCP2, resulting in slightly different hydrodynamic radii that could affect retention on size exclusion phase.

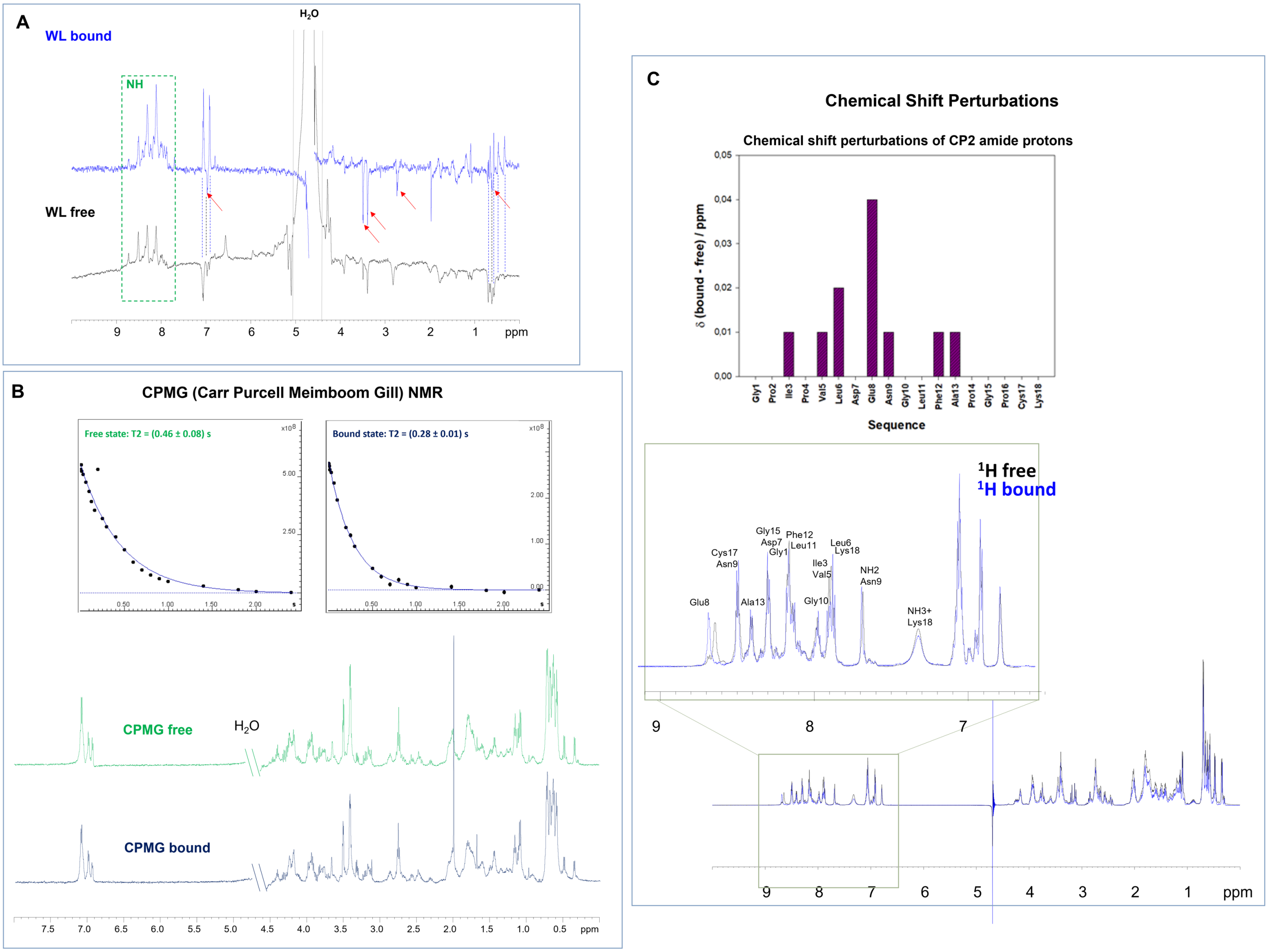

Figure S3. WaterLOGSY (WL), CPMG and 1D NH chemical shift perturbation NMR experiments on the mixture of mAb 2C7 with peptide CP2. **A, WL experiment.** In WL spectra, the change in the peak phase passing from the free to the bound state is indicative of binding. The positive signals in WL correspond to the peptide protons most involved in the binding with mAb that are also mediated by surrounding water molecules. The red arrows indicate the signals that remain negative in the bound state; and that do not interact with the antibody. **B, CPMG experiments**. Comparing the CPMG experiments of the peptide alone and in the mixture with the antibody, a slight decrease of signals in the bound state and differences in the T2 values are detected with respect to the free state. The decrease of transverse relaxation time T_2 of CP2 signals upon binding was indicative of complex formation. **C, 1D NH chemical shift perturbation.** Because of the rapid exchange regime, we observed broadened CP2 ligand resonances (or reduction of signal intensity) and a few chemical shift perturbations, which was further evidence for binding of the CP2 peptide to the 2C7 antibody. Upon mAb-CP2 interaction, changes in the chemical and magnetic environment at the peptide protein interfaces are induced, hence affecting the chemical shifts of the nuclei in this region.

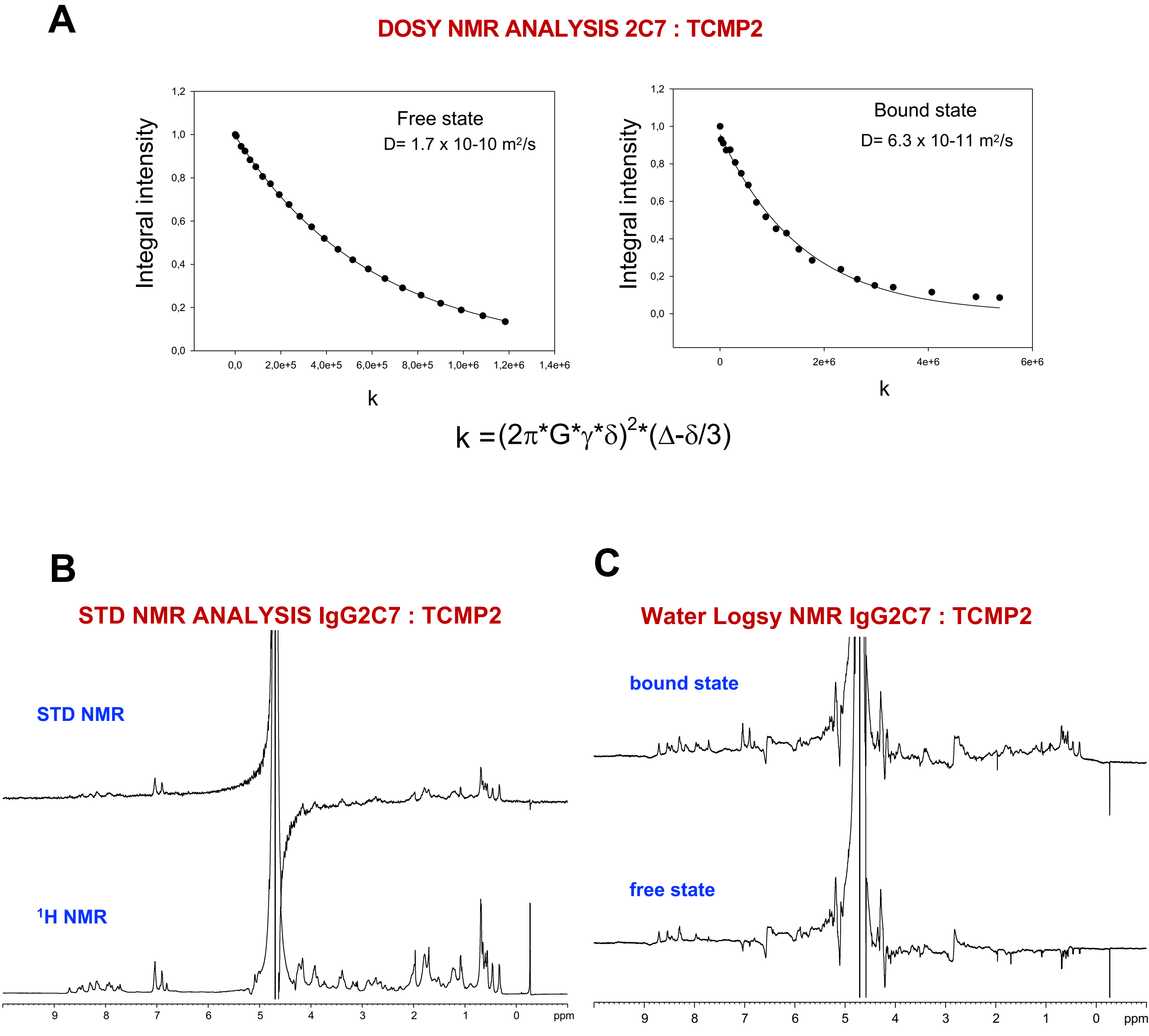

Figure S4. NMR analysis of the mixture between mAb2C7 and TCMP2. **A,** Pulsed-field gradient (PFG)-NMR measurements of the TCMP2 in absence and presence of mAb2C7 to determine the diffusion coefficients of the molecules (top). **B and C**, STD and WL NMR experiments show the molecular binding between mAb2C7 and TCMP2.

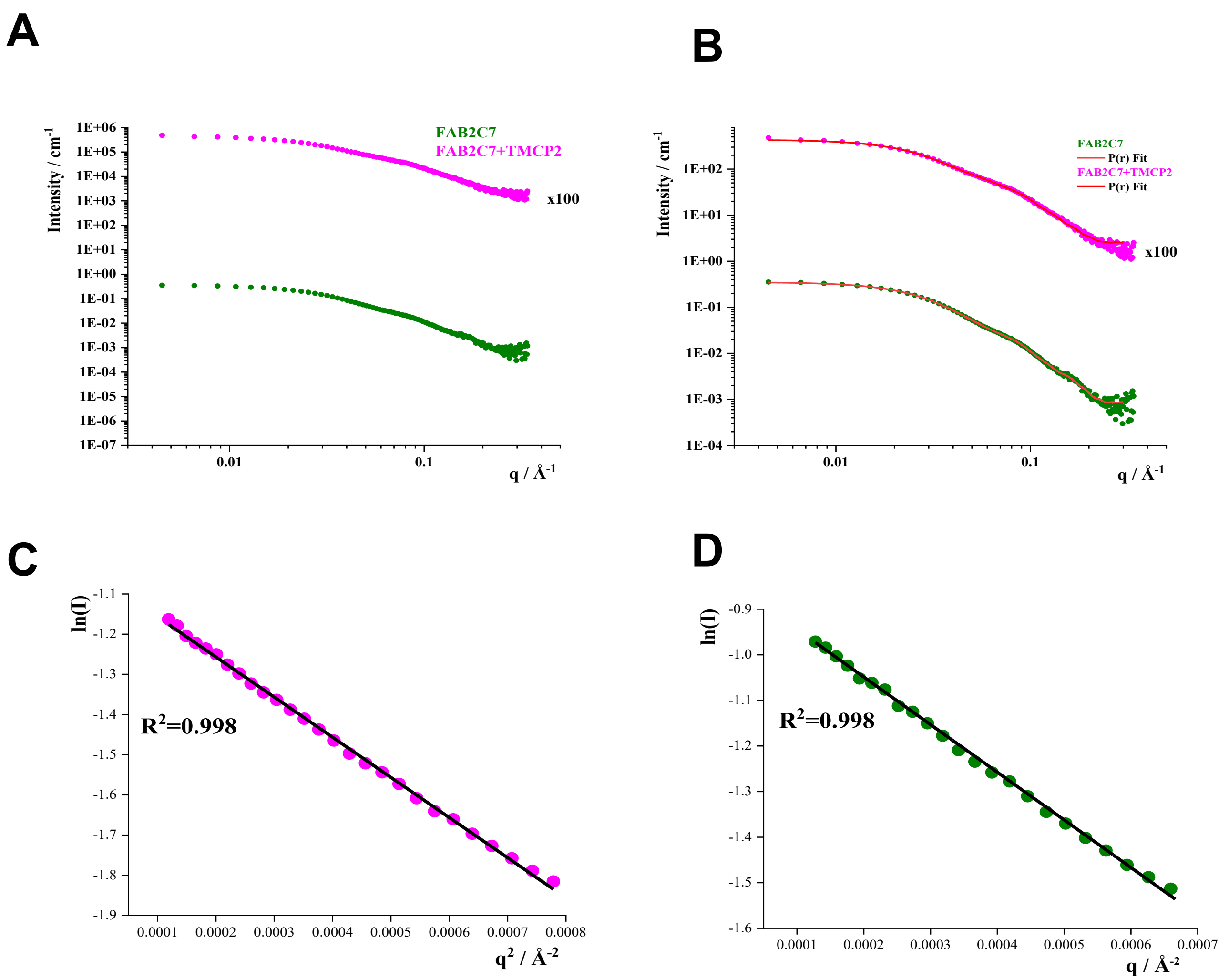

Figure S5. Small angle X-ray scattering (SAXS) experiments. **A,** SAXS profiles of Fab2C7 (green) and Fab+TMCP2 (magenta). **B,** SAXS profiles with P(r) fitting shown as red line. **C,D,** Guinier analysis of the low-q data.

A

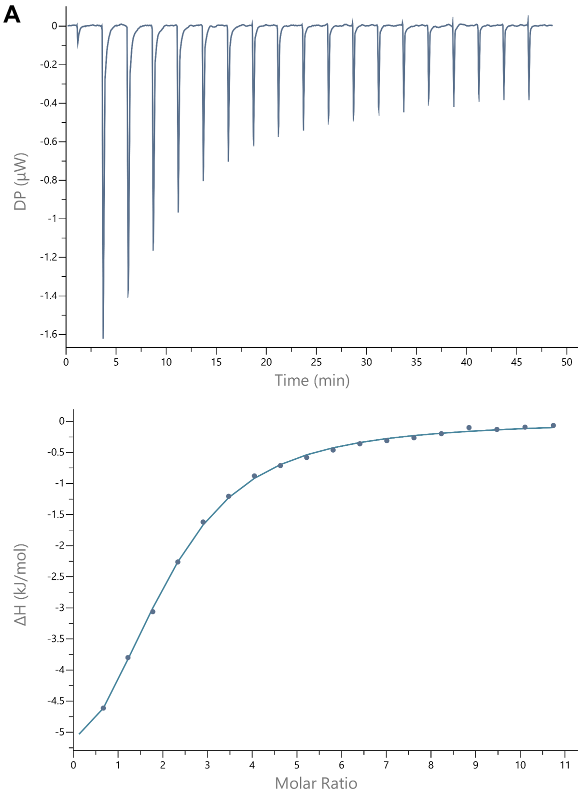

B

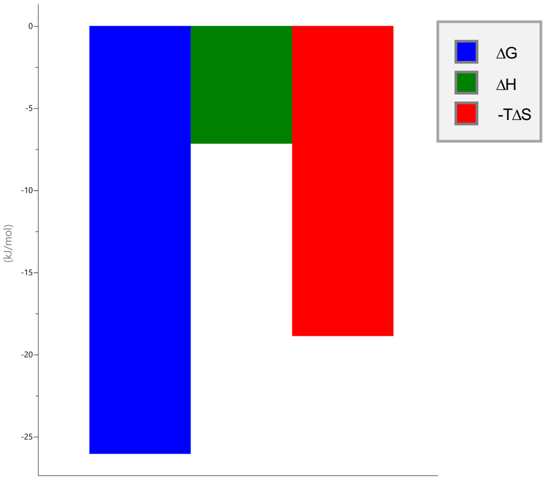

C

N (sites)	K_D [µM]	ΔG [kJ/mol]	ΔH [kJ/mol]	$-T\Delta S$ [kJ/mol]
1.89 ±4.4e-2	23.0 ± 1.85	-26.1	-7.17 ± 0.296	-18.9

Figure S6. ITC analysis. Summary of thermodynamic parameters for CP2 interacting with mAb 2C7. **A,** Baseline-leveled raw data of mAb 2C7 titration with CP2 (*upper panel*) and the resulting curve fitted using a single binding site model (*lower panel*). **B,** Histograms of the thermodynamic parameters defining the binding of CP2 to mAb 2C7; the numeric values are shown in panel **C**.

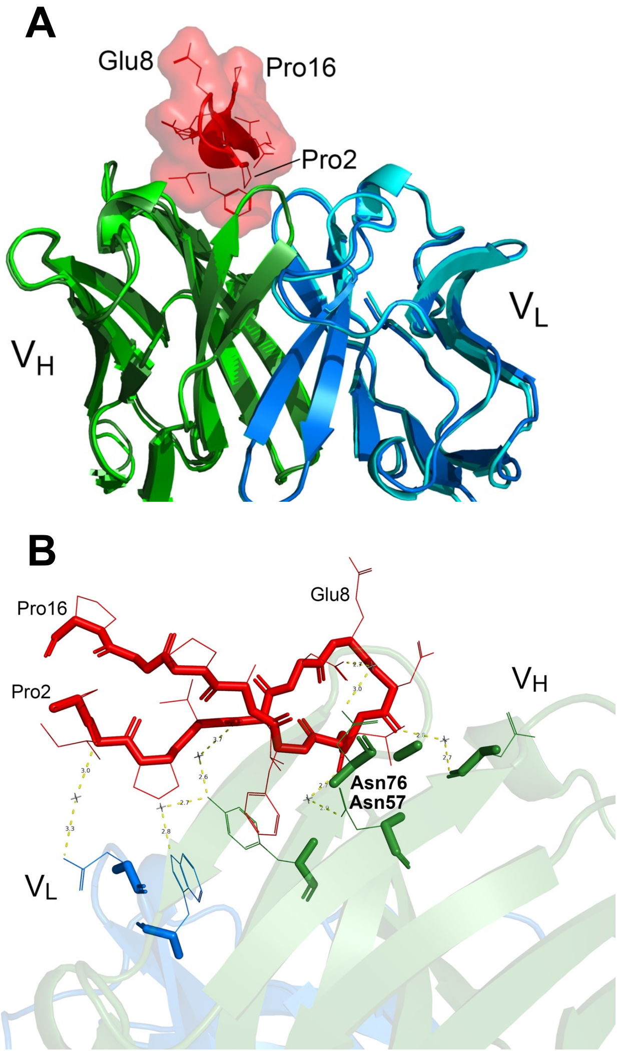

Figure S7. Structural details of Fab-peptide complex. **A,** Superposition of Fab alone and Fab-peptide complex. Fab 2C7 alone is shown in light green (VH) and aqua (VL) and the Fab from the complex is shown in dark green and blue (VH and VL, respectively). The superposition was based on the variable region domains. **B,** Ordered water molecules bridging Fab 2C7 and CP2. Seven ordered water molecules were observed making putative H bonds with both Fab and peptide chain F (shown in Figure), and six for peptide chain E (not shown). Of these, two water molecules were seen bridging the same atoms in both copies of the asymmetric unit of the crystal (chain A/C: Asn57 ND2 to F/E: Gly10 O, and A/B: Asn76 ND2 to F/E: Asp7 OD2). Distances in the two independent copies are given in Table S4.

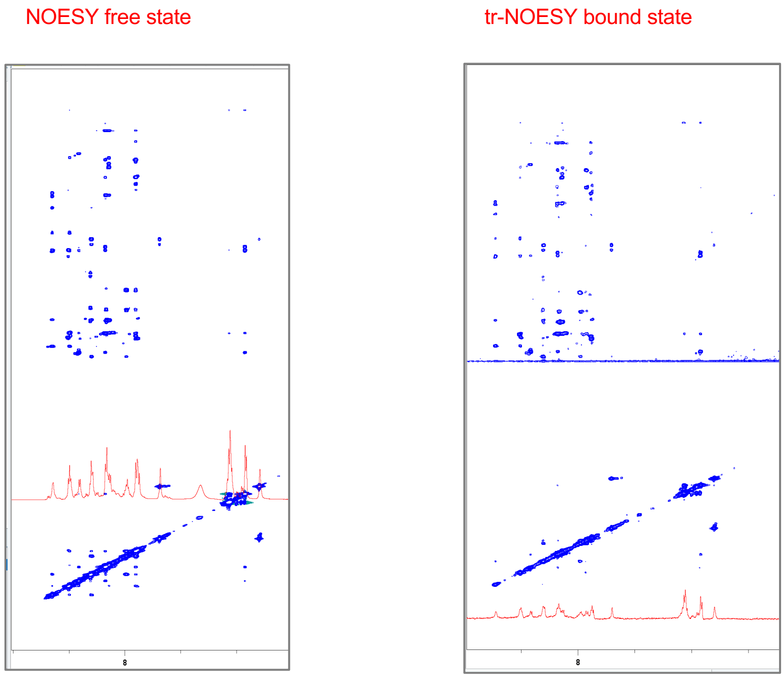

Figure S8. NOESY and transferred-NOESY (tr-NOESY) NMR spectra to study the conformational behavior of CP2 peptide alone and bound to the antibody.

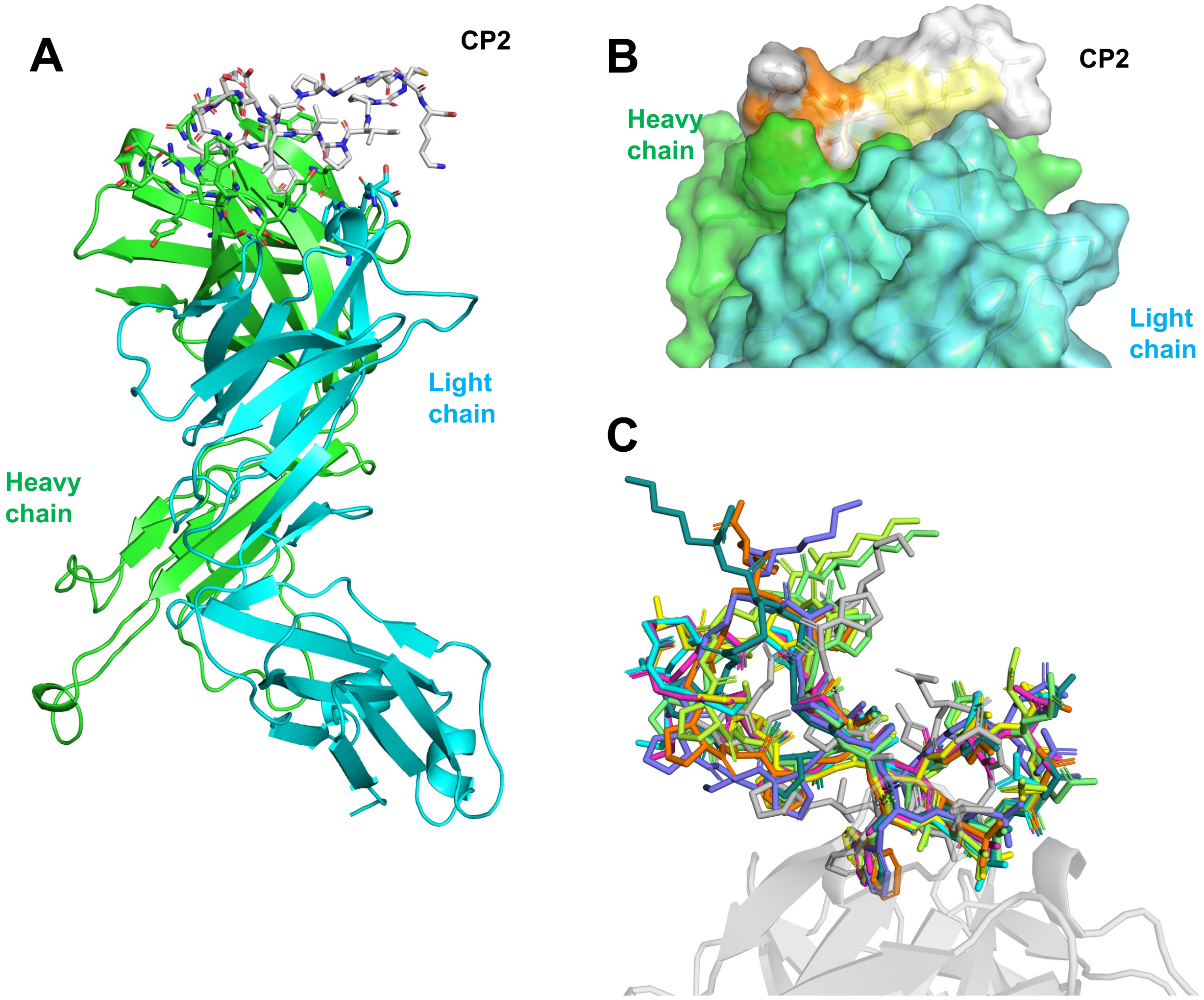

Figure S9. 3D views of Fab2C7 and CP2 complex obtained by NMR and MD. **A,** The most populated conformation obtained from the MD cluster analysis. **B,** CP2 in its bioactive conformation, with the surface colored according to the STD effects (from the highest in red to the lowest in yellow), in complex with Fab2C7. **C,** Superimposition of the different poses of Fab2C7 in complex with CP2 in its bioactive conformation obtained from MD simulation.

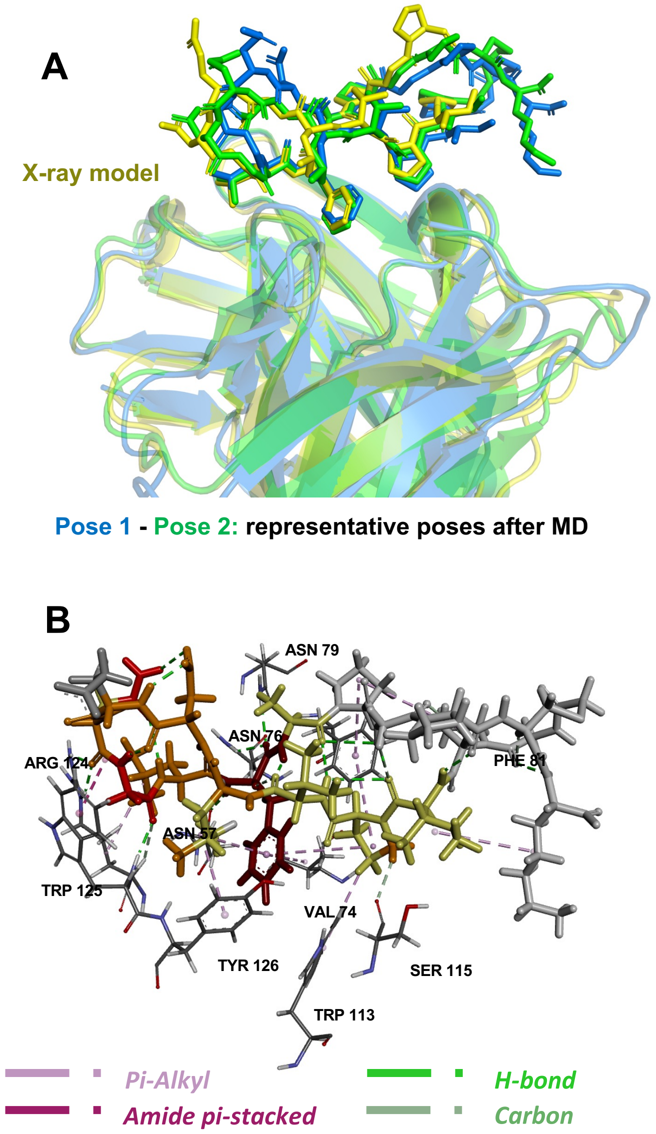

Figure S10. A, MD representative conformations of Fab2C7 in complex with CP2 in its bioactive conformation as determined by NMR analysis (blue and green) superimposed to the X-ray structure (yellow). **B,** Different 3D views of the most populated pose of Fab2C7-CP2, obtained from the MD cluster analysis, according to the STD edit code and tr-NOESY analysis.

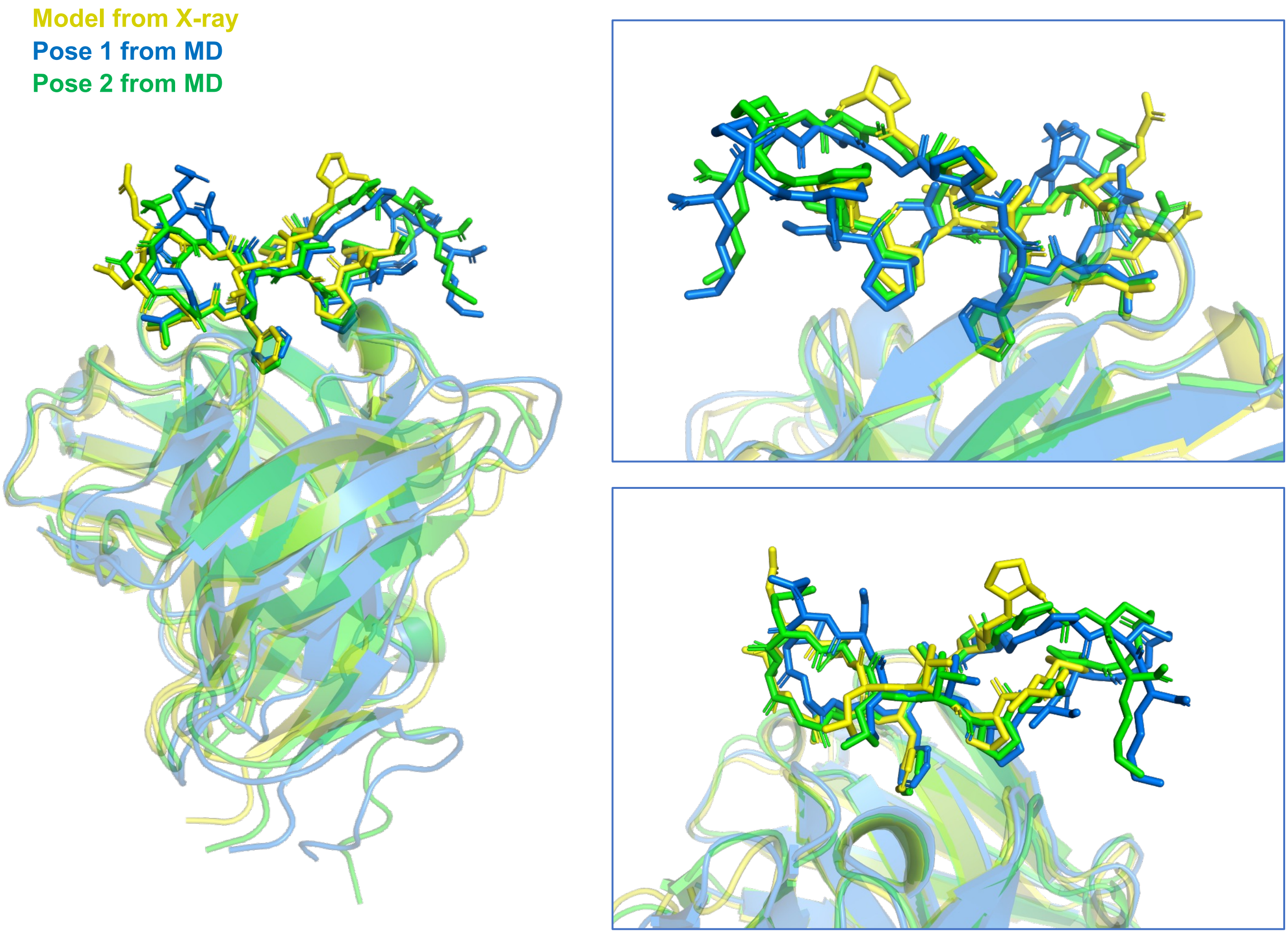

Fig. S11. Different views of Fab2C7-CP2 models obtained by MD simulations superimposed on the X-ray structure.

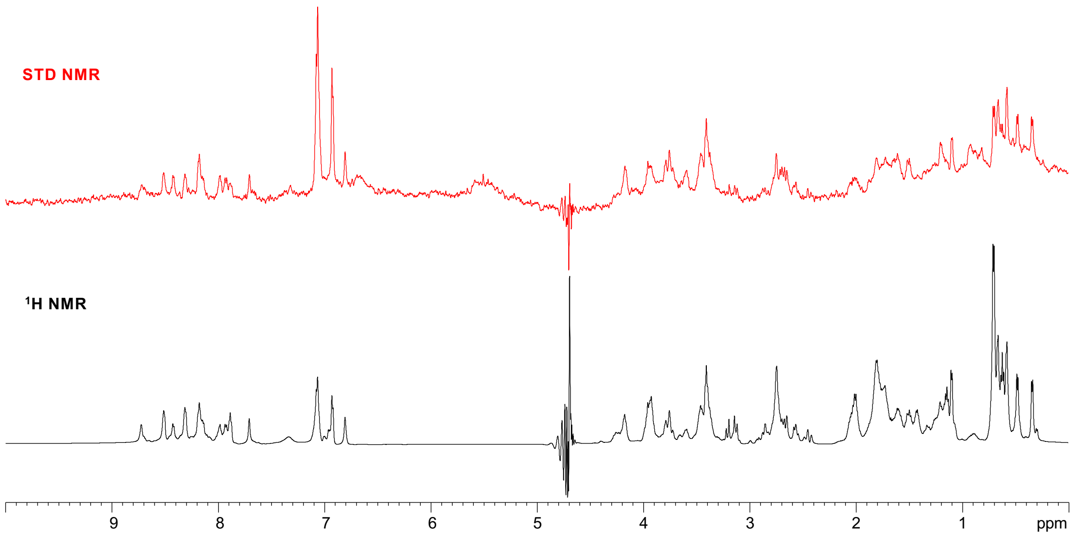

Figure S12. NMR spectra of Fab 2C7 in complex with CP2. Comparable STD NMR and tr-NOE results were obtained for the interaction between Fab 2C7 and peptide CP2.

Fab 2C7 IgG heavy chain, Fd fragment

EVQLQQSGPELVKPGSSVKISCKGSGYTFTDYNMEWVKQSHGKSLEWIGVINPNNRFTSYNQNFRG
KATLTVDKSSSTAYMDLRSLTSEDSAVYFCAGSRWYQYDYWGQGTTLTVSSASTKGPSVFPLAPSS
KSTSGATAALGCLVKDYFPEPVTVSWNSGALTSGVHTFPAVLQSSGLYSLSSVVTVPSSSLGTQTY
ICNVNHAPSNTKVDKKVEPKSCD

Fab 2C7 Ig, lambda light chain

QVVVTQESALTTSPGETVTLTCRSSTGAVTTSNYANWVQEKPDHLFTGLIGGINNRAPGVPARFSG
SLIADKAALTITGAQTEDEAIYFCALWYSNHWVFGGGTKLTVLGQPKAAPSVTLFPPSSEELQANK
ATLVCLISDFYPGAVTVAWKADSSPVKAGVETTTPSKQSNNKYAASSYLSLTPEQWKSHRSYSCQV
THEGSTVEKTVAPTECS

CP2 mimetic peptide

GPIPVLDENGLFAPGPC

Figure S13. Amino acid sequences of Fab 2C7 heavy chain (Fd fragment), light chain (lambda) and CP2.
